# Psychoeducation Group for Depression (PEG-D): Study protocol for a prospective, randomized, single-blind, crossover trial

**DOI:** 10.1371/journal.pone.0329006

**Published:** 2025-08-08

**Authors:** Adriana Munhoz Carneiro, Sara Lisboa Teodoro Silva, Fernando dos Santos Fernandes, Jainan Rodrigues Barretto, Tatiane Rodrigues de Brito, Lilian Hupfeld Moreno, Fernando Cordeiro Pimentel, Bruna Alcaire Jorge, Leonardo Afonso dos Santos, Matheus Rassi Fernandes Ramos, Ricardo Alberto Moreno

**Affiliations:** 1 Mood Disorders Unit, Faculty of Medicine FMUSP, University of São Paulo, São Paulo, Brazil; 2 Department and Institute of Psychiatry, Service of Interdisciplinary Neuromodulation (SIN), University of São Paulo Medical School, São Paulo, Brazil; Jouf University College of Nursing, SAUDI ARABIA

## Abstract

**Background:**

Considered a leading contributor to the global health burden, depression is characterized by its multifaceted nature. Recognizing the limitations of pharmacological treatments, non-pharmacological alternatives have been suggested to enhance full remission and increase quality of life.

**Aim:**

To contribute to this field, we aim to develop and investigate the efficacy of a short psychoeducational program as an adjunctive treatment for depression.

**Methods/design:**

We propose a single-center, prospective, crossover, single blind, randomized controlled trial, with 190 patients diagnosed with Major Depressive Disorder-MDD (DSM-5-TR), and moderate or intense severity by Hamilton Depression Rating Scale -HAMD-17. Participants will be randomly assigned to one of two arms, Treatment as usual (TAU) + Psychoeducation (n = 96) or TAU (n = 96). The psychoeducational program will consist of six weekly group sessions (6–10 participants) with each session lasting 90 minutes, covering information and techniques for identifying and coping with depression. Reduction in depressive levels, measured by the Beck Depression Inventory-BDI-II, and an increase in their level of knowledge on depression will be considered our primary outcome. Secondary outcomes, such as improvement in functionality and quality of life, will be measured by the Global Functioning Rating Scale (GAF), Clinical Global Impression Scale, and cognitive and behavioral factors by the Cognitive and Behavioral Avoidance Scale-CBAS, and Thoughts Scale Depressants-DTS (EPD).

**Discussion:**

Developing and validating a structured psychoeducational program for depression is crucial for understanding the contribution of psychoeducation for depression and to facilitate the replication of findings, if positive. If our program demonstrates efficacy-superiority compared to TAU treatment, it might present as an interesting, cost-effective alternative as an adjunctive treatment. An expansion to other modalities, including open groups, euthymic patients, and online formats, might be considered, depending on our results.

**Trial registration:**

NCT06467474

## Background

Depression is a highly prevalent mood disorder, classified as the most prevalent health-burden mental disorder worldwide [1]. In the context of treatment, psychosocial treatments provide benefits and are indicated as a standard treatment [2]; however, many patients face difficulties adhering to recommendations [3].

Treatment adherence is directly related to a good treatment response [4,5]. Understanding the pharmacological treatment plan, as it is related to patient concerns about medication (mechanism of action, side effects, and duration), helps patients to comprehend more about their treatment and what to expect. Also, it is important for patients to understand which factors are capable of influencing treatment efficacy, as patient hesitancy and knowledge gaps can hinder treatment effectiveness; providing treatments that address these issues is essential [6].

Among those treatments, psychoeducation (PE) is a psychosocial treatment that focuses on promoting patient education and training about the disease and related aspects, standing out as an auxiliary strategy to other treatments [7]. This approach has proven to be a promising option due to the feasibility of being implemented in different formats, including individual or group sessions, and in different modalities, which can be offered by different health professionals or even self-guided [8].

Although PE is a method used and referenced in the literature over time [9,10] and well established for some disorders, it still needs further clarification, especially in depressive disorders. The results on psychoeducation have primarily focused on measures of the clinical course of the disease [11], while few have examined whether the information provided has been effectively absorbed by the target audience. Moreover, the mechanisms through which psychoeducation generates its benefits remain largely unexplored [12]. The reason that we developed and decided to investigate the efficacy of a new psychoeducational program for depression, named Psychoeducational Group – Depression PEG-D.

### Objectives

We aim to investigate the efficacy of a new psychoeducational program to reduce depressive symptomatology in patients diagnosed with MDD in moderate/severe symptomatology at baseline. We also have as a primary outcome the impact of the level of acknowledgement from PE intervention on MDD symptoms. Secondly, we will investigate the impact of the treatment on functionality, quality of life, and cognitive and behavioral responses. By applying this protocol, we expect to understand the specific contribution of a psychoeducation model designed in a randomized-controlled way, and to establish a replicable program that may be generalized and adapted for diverse patient populations and delivery methods. We hypothesize that participants undergoing the PEG-D intervention will demonstrate greater improvement in depressive symptomatology, as well as a higher level of disease-related knowledge, compared to those receiving only treatment as usual (TAU). Moreover, by incorporating secondary measures of cognitive and behavioral avoidance and dysfunctional beliefs, we expect to gain insight into possible mechanisms through which psychoeducation exerts its effects.

### Trial design

The Psychoeducational Group for Depression (PEG-D) is a single-site, prospective, randomized, controlled clinical trial with a crossover design and single blinding. Participants will be randomly assigned to one of two groups: the clinical group, which will receive Treatment as Usual (TAU) combined with the psychoeducational intervention (PEG-D) from the beginning; and the control group, which will receive TAU only during the first six months. After this initial phase, control group participants will also receive the psychoeducational intervention in addition to TAU. This design allows for the evaluation of psychoeducation at different time points while ensuring that all participants ultimately receive the intervention. The use of TAU as a comparator was chosen to reflect real-world clinical practices in the management of depression in specialized care settings. This choice enables the assessment of whether the psychoeducational intervention provides additional benefits beyond standard care, such as pharmacotherapy and regular psychiatric follow-up [2]. The protocol was based on SPIRIT guidelines [13] (S1 Fig).

## Methods

### Study setting

The study will be conducted at the Mood Disorders Outpatient of the Faculty of Medicine of the University of São Paulo (FMUSP), São Paulo, Brazil (CAAE: 76212623.1.0000.0068)..

### Eligibility criteria

Considering our aim to investigate the efficacy of PEG-D for improvement in the treatment of MDD, our eligibility criteria will consider for inclusion patients of both sexes, aged between 18 and 65 years. The diagnosis of Major Depressive Disorder (according to DSM-5-TR [14]) will be made using the Structured Clinical Interview for DSM Disorders (SCID) [15] and confirmed by a clinician. Patients will be considered eligible if considered in moderate to severe depressive episode, measured by the Hamilton Rating Scale for Depression (HAMD-17) (scores between 14 and 23) [16,17].

Considering that this is a local study, participants must be able to participate in the in-person sessions required for the study protocol. Participants will be excluded if they have unstable, serious clinical or neurological diseases, defined as progressive or unpredictable conditions that could significantly worsen during the study period, potentially compromising participant safety or data integrity. This exclusion criterion will be assessed by a trained clinician during the screening phase. Postpartum depression or other types of depressive disorders (such as disruptive mood dysregulation disorder, premenstrual dysphoric disorder, substance/medication-induced depressive disorder, depressive disorder due to another medical condition, other specified depressive disorder, and unspecified depressive disorder); patients with active psychotic symptoms, suicidality risks (score >2 in HAM-D item 3), or already undergoing some other treatment (pharmacological or non-pharmacological) for MDD in which the washout is not possible will also be excluded. Other exclusion criteria include having a serious or unstable medical condition, including cardiovascular, hepatic, endocrinologic, neurological, or renal conditions.

**Fig 1:**
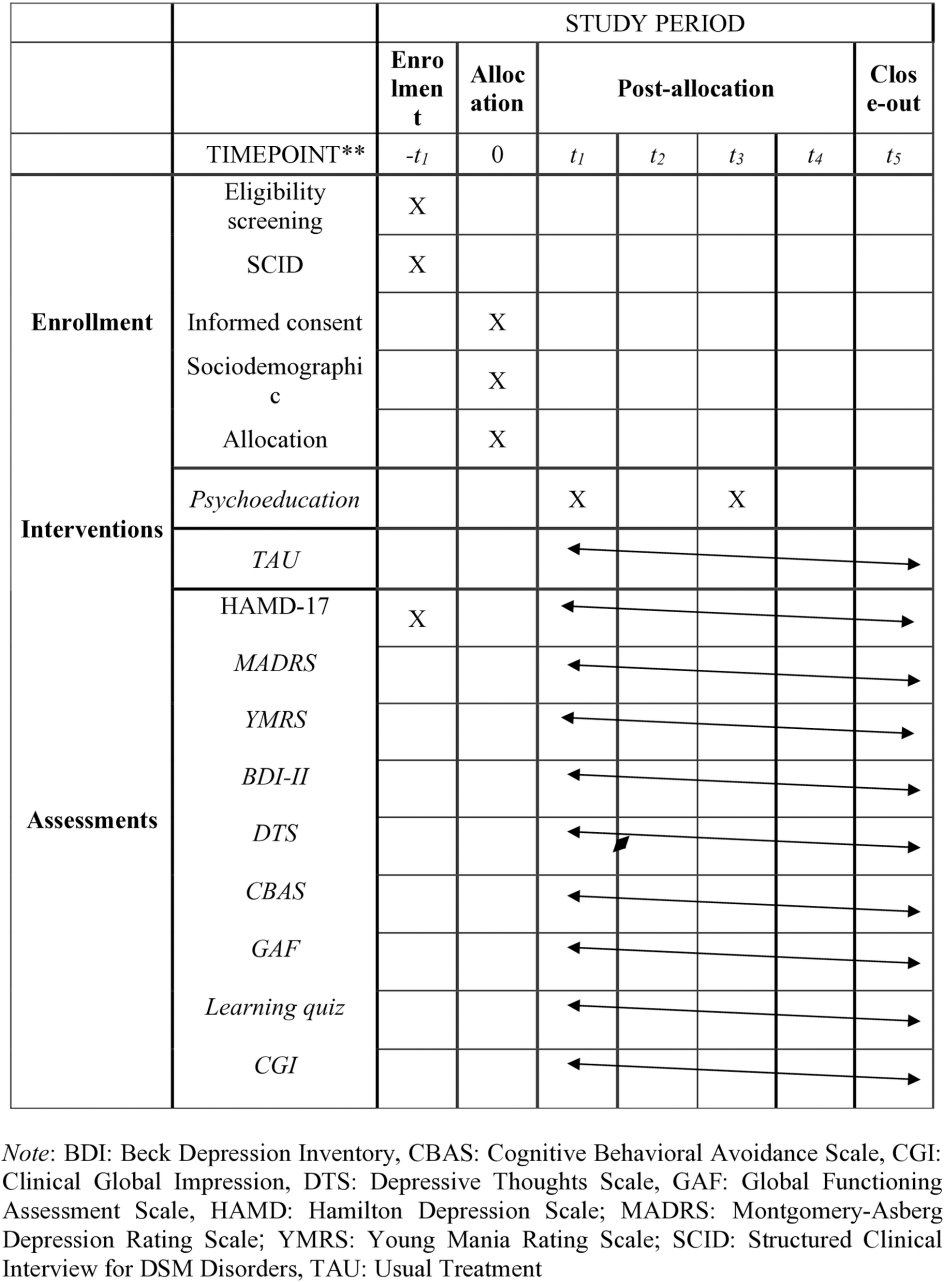
Schedule of enrolements, intervention and assessments. Note: BDI: Beck Depression Inventory, CBAS: Cognitive Behavioral Avoidance Scale, CGI: Clinical Global Impression, DTS: Depressive Thoughts Scale, GAF: Global Functioning Assessment Scale, HAMD: Hamilton Depression Scale; MADRS: Montgomery-Asberg Depression Rating Scale; YMRS: Young Mania Rating Scale; SCID: Structured Clinical Interview for DSM Disorders, TAU: Usual Treatment.

Clinically significant abnormalities on laboratory or ECG exams or those which, in the investigator’s opinion, indicate a serious medical issue, require a major intervention, or may interfere with the antidepressant treatment, are also factors for exclusion. Participants receiving psychotropic drug treatment will undergo a washout period before entering the PEG-D trial. The washout period established is one week for antidepressants, mood stabilizers, and most antipsychotics, except clozapine, which requires a 4-week (28-days) washout period along with fluoxetine [18,19] (**Fig 1**).

### Interventions

**Fig 2:**
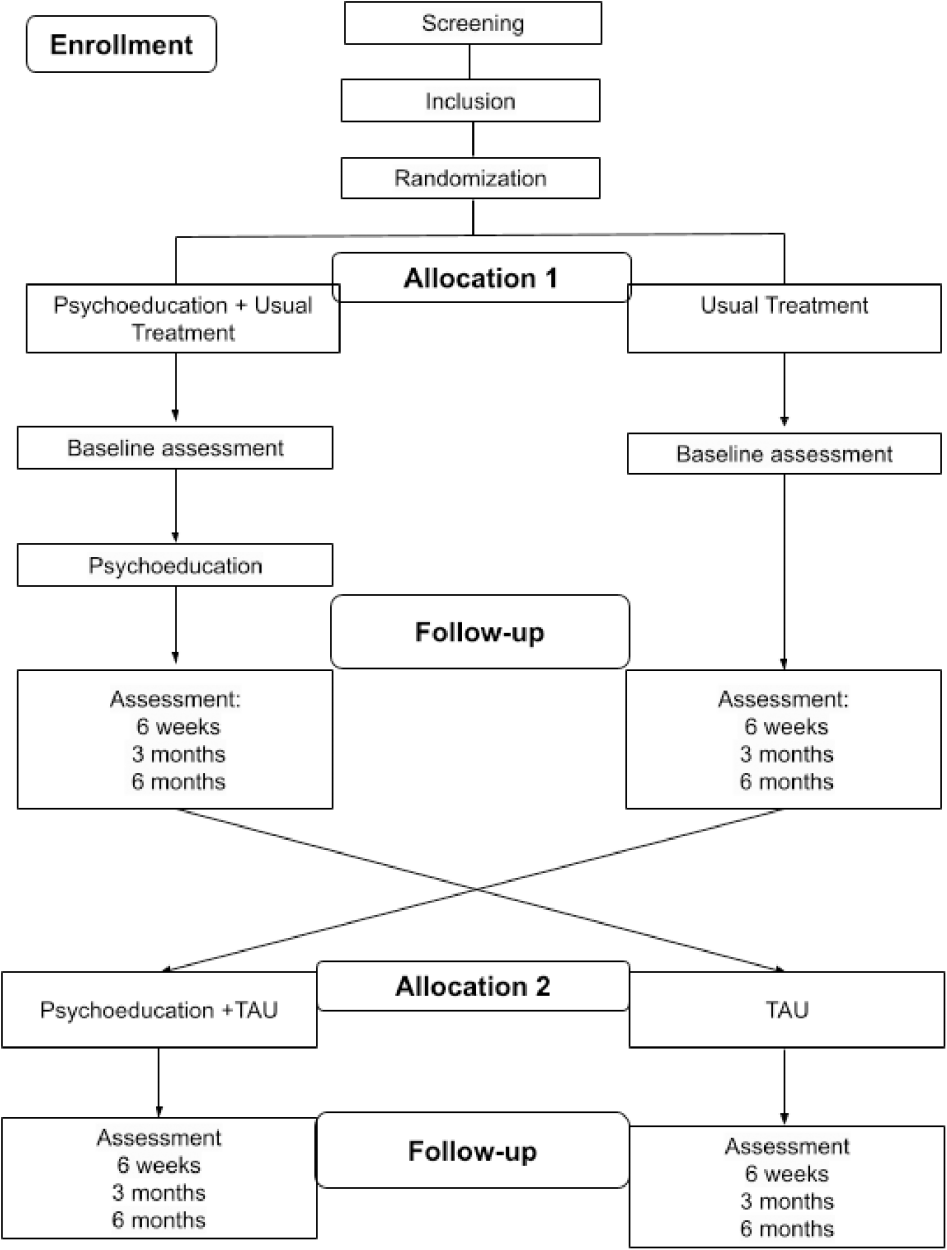
Flow diagram of PEG-D Study. Note: TAU: Treatment as usual.

**Fig 3:**
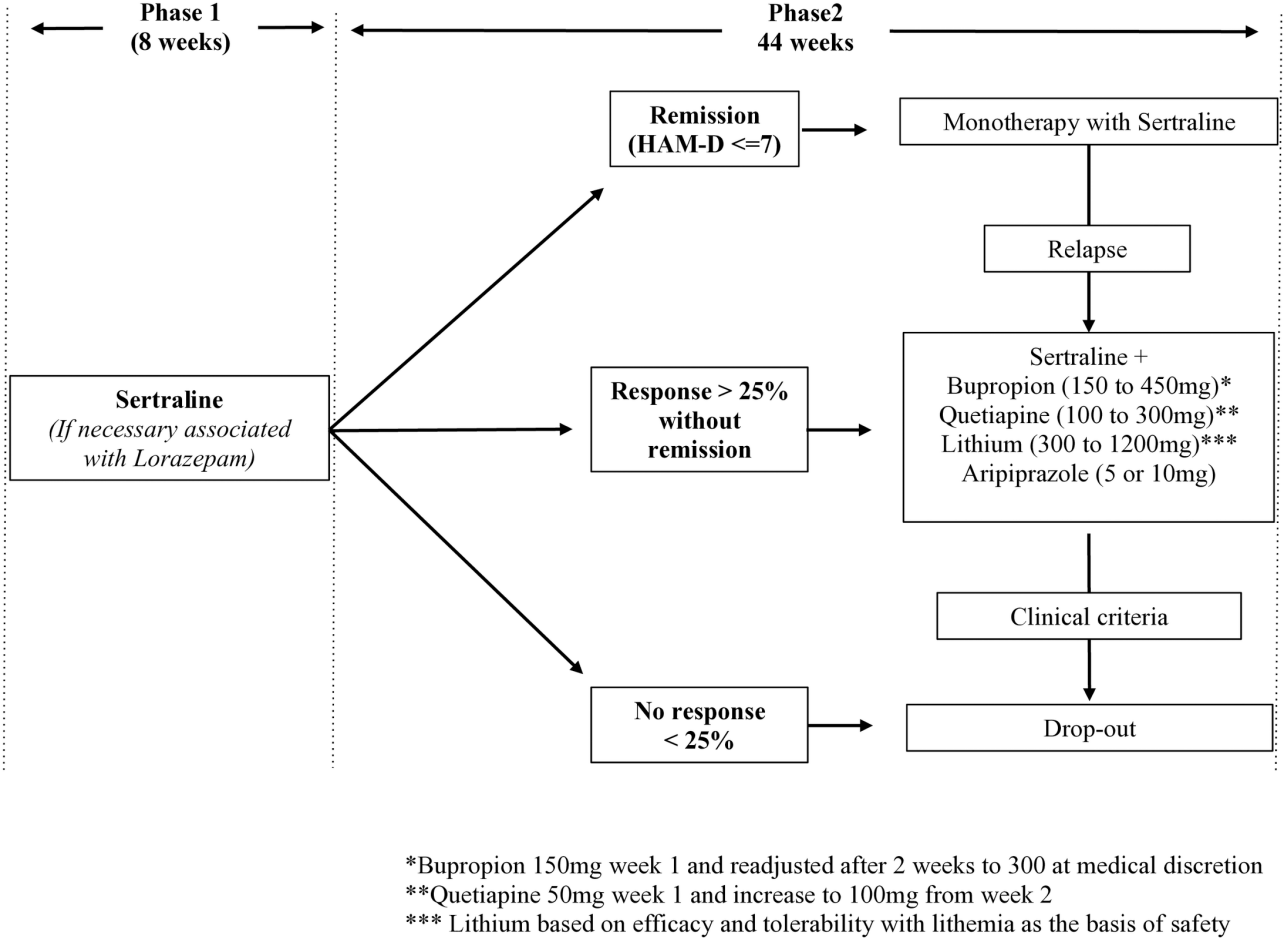
Flowchart-Treatment as Usual (TAU).

The intervention will begin one week after randomization, and treatment will last for 12 months (**Fig 2).** Our intervention is divided into the following groups:

*Treatment as usual (TAU):* Patients will start with 50 mg of Sertraline (SSRI), being adjusted according to the patient’s profile. Dosage adjustments will be made according to scores on the HAM-D and the Udvalg for Kliniske Undersøgelser (UKU) side effect rating scale [20]. After eight weeks, if the patient experiences remission, they will continue on monotherapy with Sertraline. The association with Lorazepan will also be allowed in any study phase and Buproprion, Quetiapine, Lithium and Aripiprazole will be added only in case of <25% of treatment response. Those with >25% of treatment response will be discontinued from the study (**Fig 3).**

*Psychoeducational program for Depression -PEG-D:* The PEG-D is a 6-weekly program that aims to provide guidance and psychoeducational support in order to enhance treatment adherence. Topics were defined after a systematic review and meta-analysis conducted by our group [21] and follow the guidelines provided by the Canadian Network for Mood and Anxiety Treatments (CANMAT) [2]. This way, we will not consider any specific psychological theory, but the most important topics related to previous studies in which PE was valid as an adjunctive psychosocial model (**Table 1**).

**Table 1:** Psychoeducational program content.

Topic	Content
Depression: signs and symptoms	Nature of depressive disorder, signs, and symptoms
Causes and treatment	Biological, psychological, and environmental causes of depression and an overview of pharmacological and non-pharmacological approaches
Identifying risk factors and crisis management	Factors that hinder depression improvement, crisis management strategies, and dealing with new episodes.
Benefits of a healthy lifestyle	The impact of habits, focusing on areas like diet, physical activity, and stress management in depression
Problem solving	Guidelines for an assertive communication and how to communicate decisions
Practical strategies	How to differentiate the interpretation of facts and behavioral activation strategies that contributes to cope with depression

* *Note*: our table illustrate the topics of our 6-weekly program. Sessions will be in a group format, lasting 90 min each, in an open modality, in which new participants can start the program in any session.

The complete program will last six consecutive weeks, with sessions lasting 90 minutes each. The intervention will be applied in a standardized manner regarding content and objectives, but will allow adaptations according to the group’s needs, considering individual aspects that may influence participation and engagement. Participants’ adherence to both TAU medical consultations and psychoeducation will be monitored by the professional team member responsible for managing their treatment.

Participants will receive a booklet containing the content of the six sessions, with explanatory texts, images, and tables on the first day of their PE. During the sessions, slides with guiding topics will be used to structure the discussion, and homework tasks to generalize the content will be assigned. The intervention will be conducted in a group format, with in-person sessions structured in three main parts, as follows: (1) initial questions regarding participants’ understanding of the topic, (2) theoretical explanation provided by professionals, (3) discussion and practical application of the concepts covered. The sessions will take place in hospital outpatient rooms, equipped with audiovisual resources for slide projection, and will follow an open modality, in which new participants can start the program in any session.

The intervention will be led by a primary therapist and a co-therapist for additional support, and they should have a background in Psychology or other Mental Health areas (i.e., psychiatry, nursing). Before participating as co-therapists, they will receive an 20-hour training (4-hours of theoretical training and more 12 hours as observer training, by participating from two PE groups).

Fidelity of the psychoeducation delivery: Inter-rater consistency is monitored every 6 months by professionals receiving ongoing training to ensure the quality and uniformity in the application of the interventions and evaluations. In the PE group, for example, the professional acts as a co-therapist in at least 12 PEG-D sessions (corresponding to approximately 2 rotations) before becoming the primary therapist.

Criteria for discontinuation: The criteria for interrupting the study are more than two absences during treatments; initiation of other treatments for depression during our study; non-response to the use of Sertraline in the initial eight weeks; HAMD score >2 on item 3; presence of symptoms of mania at any time during treatment. For this issue, we will consider a score equal to or higher than seven in the Young Mania Rating Scale (YMRS ≥7) [22].

Adherence plan: To increase adherence to interventions, participants will receive a text message throughout the week of appointments containing specific scheduling details. Follow-up messages will be sent to monitor their well-being and treatment adherence. For patients who are receiving psychoeducation, follow-ups may be done in person.

### Outcomes

#### Primary outcomes.

- Beck Depression Inventory-II (BDI-II) [23]: Selected as the primary outcome, the BDI-II, is a self-reported measurement aiming to gather a more broad information regarding affective and cognitive symptoms. It consists of a 21-item four-point *Likert* scale, where the higher the score, the greater the intensity of depression in the last 14 days. The Brazilian version validated for Brazil by Gorenstein et al. [24] has an internal consistency (Cronbach’s alpha) between 0.89 and 0.92 and it also presents sensitivity to change, being suitable for clinical and research contexts. By using this measurement as the primary outcome, we believe it will provide not only more information about participants’ depressive symptoms and severity but also insight into their participation in our crossover study.
- Learning: To evaluate knowledge and generalization of PE contents, we developed a questionnaire specifically for this study. Based on the PE contents, it is organized in two parts and will serve as our learning assessment measure, primarily for qualitative data. The first section (structured section) comprises eight multiple-choice items, in which participants should rate the level they agree with some statements, followed by two open questions, in which participants should describe their symptoms and how depression affects their daily life, and finally. The second part is a non-structured section, with a case study, in which participants should respond to questions regarding treatment and symptom recognition; and a list of 15 symptoms that they should read and mark which one corresponds to a depressive episode. We expect that with this questionnaire to understand how patients are assimilating the information received through PE and if they will have some difference in knowledge and understanding compared to baseline.

#### Secondary outcomes.

- Cognitive Behavioral Avoidance Scale (CBAS) [25] assesses avoidance styles commonly seen in individuals with depression and includes 31 items across four factors: behavioral/social, cognitive non-social, cognitive social, and behavioral non-social avoidance, with reliability coefficients ranging from 0.75 to 0.80 in the original Canadian version.
- Depressive Thoughts Scale (DTS) [26] was developed based on Beck’s cognitive triad and items from widely used instruments assessing depression-related beliefs. Items were created by the authors and selected from translated scales that reflected beliefs of incapacity, self-blame, guilt, worthlessness, and low self-esteem. The version includes 26 items across two factors: low self-esteem/hopelessness (16 items) and functionality in relationships (10 items). The scale showed high reliability, with Cronbach’s alpha of 0.93 for the total score, 0.93 for Factor 1, and 0.89 for Factor 2. Higher scores indicate more dysfunctional depressive thoughts.
- Global Functioning Rating Scale (GAF) will be used to assess participants’ overall psychological, social, and occupational functioning at the time of evaluation. The GAF is a numeric scale ranging from 0 to 100, with higher scores indicating better functioning. In a prior study [27] with outpatients with depression, mean GAF scores showed modest interrater agreement (r = 0.26). GAF scores were significantly associated with symptom severity (MADRS, BDI-II) and functional outcomes (SF-36), supporting its validity as a global measure of functioning.
- Clinical Global Impression Scale – CGI is a clinician-rated global assessment tool that considers all available information, including patient history and functional impact of symptoms. It includes two components: the CGI-Severity (CGI-S), which rates illness severity from 1 (normal, not ill) to 7 (among the most extremely ill), and the CGI-Improvement (CGI-I), which assesses overall improvement since treatment initiation, ranging from 1 (very much improved) to 7 (very much worse). Effect sizes between CGI-I and CGI-S ratings were large, with significantly greater changes in CGI-I. Interrater agreement for CGI-S was low (ICC = 0.37; CI 0.15–0.59), while for CGI-I it was moderate (ICC = 0.65; CI 0.47–0.80), demonstrating good reliability for the assessment of clinical improvement [28].

#### Clinical profile assessment.

- Hamilton Depression Rating Scale [16], we utilized the validated version [17]: it is a hetero-evaluation scale, used to assess the severity of the depressive episode in patients with mood disorders; however, it is not a diagnostic instrument for identifying depression. The assessment of depressive mood considers the presence/absence of symptoms over the last week. It has mostly cognitive and vegetative symptoms, evaluating a smaller number of items of social, motor, anxiety, and mood factors, and allows the classification of mild, moderate, or severe depression. The reliability of the HAM-D scale is cited only in its international version, which varies from 0.83 to 0.94.
- Montgomery-Asberg Depression Rating Scale – MADRS [29]: The MADRS is a hetero-evaluation scale developed to be sensitive to changes in symptoms throughout treatment. It consists of 10 items, scored from 0 to 6, with two of them rated by the observer. In this study, the validated Brazilian Portuguese version [30] will be used. The scale shows good sensitivity to different levels of depression severity, as well as good internal consistency and relative validity compared to the HAMD [31]. Müller and Szegedi [32] showed that increasing the reliability from 0.5 to 0.8 raises the test’s ability to detect significant differences between two treatments from 51% to 71%, and the cutoff points adopted from another study [33] are 0–8 (remission), 9–17 (mild), 18–34 (moderate), and >35 (severe).
- Young Mania Rating Scale - (YMRS) [34]: scale composed of 11 items, scored from 0 to 4 and from 0 to 8 (irritability, speech, thought content, and disruptive-aggressive behavior, scored twice to compensate for the patient’s lack of cooperation). It is the most widely used scale in clinical studies with manic patients, with a high inter-rater reliability index (around 0.93 for the total score and between 0.66 and 0.92 for individual items). The version used [22] was adopted, with a cut-off point <7 as indicative of significant symptoms of mania/hypomania.

### Participant timeline

All patients will be followed for 12 months and assessed at five time points: baseline (*t*_*1*_), after six weeks at the end of the psychoeducational program (*t*_*2*_), at 3 months (*t*_*3*_), 6 months (*t*_*4*_), and at the 12-month closeout (*t*_*5*_) (**Fig 1**).

### Sample size

The number of participants for the study was established through sample calculations using the G*Power software [35]. A sample size calculation was conducted for a repeated measures MANOVA with a between-subjects factor, considering a moderate effect size (f = 0.25), significance level of 0.05, statistical power of 0.95, two groups, five repeated measurements, and a correlation among repeated measures of 0.70. The F test was used with the O’Brien-Shieh algorithm and Pillai’s Trace statistic (Pillai V = 0.0759878), resulting in a required total sample size of 160 participants, with a noncentrality parameter (λ) of 13.16, critical F value of 3.90, 1 numerator degree of freedom, and 158 denominator degrees of freedom, ensuring sufficient power (0.9500442) to detect the proposed effect. To account for potential participant attrition, we have increased this number by 20% of the attrition rate, to ensure sufficient power for the study despite potential dropouts, resulting in a total sample size of 192 participants.

### Recruitment

Recruitment starts at month 4, and we expect to perform an interim analysis at month 26. A specific plan for dissemination will be developed, including institutional newsletters, press and media releases, flyers, and training courses throughout the national context to promote the implementation of the intervention on a large scale in the last 12 months. Moreover, users and carers will be involved in the process of dissemination by organizing thematic conferences to present the characteristics of the intervention. In order to broaden our sample, we opted for a mixed recruitment method. Participants will be recruited through invitation letters and posters on local websites, as well as from the community through flyers and advertisements on digital media. Recruitment is divided into three main phases (P), as described above:

- P-1[Screening]: Interested participants will be redirected to a site with a brief questionnaire, and those who match the criteria will be contacted by phone and redirected to a pre-trial interview.
- P0 [Pre-trial phase I]: In the pre-trial interview, participants will be evaluated by HAMD, if a depressive episode is found with scores (14 and 23), the Structured Clinical Interview (SCID) will be continued and applied by a trained psychologist or psychiatrist. If those criteria are confirmed, a detailed psychiatric consultation with a psychiatrist will be conducted.
- P1 [Pre-trial phase II]: to gather more information regarding the diagnosis, other diseases, and exclude any possibility of confoundings.

After this step, participants will be enrolled into the study and randomly assigned to one of the two available treatment as usual plus PEG-D or Treatment as usual arms. After six months, patients will crossover. For more information, see the flowchart (**Fig 2**).

### Assignment of interventions

#### Allocation.

All patients who give consent to participate and who meet the inclusion criteria will be randomized. Participants will be randomly assigned to the intervention or control group through simple randomization (1:1) via the website https://www.randomizer.org, and this list will be imported to the REDCap software [36]. By using REDCap randomization, we ensure confidentiality by concealing the random distribution list, maintaining an unalterable randomization sequence, and permitting only controlled access to the randomization tool to authorized researchers not directly involved in the data collection. The same platform (REDCap) will be used to store the evaluation data.

To investigate the effectiveness of the PEG-D program, professionals responsible for evaluating depression severity, consultations, and assessments will be conducted throughout the study in a blind manner. Participants will be advised to avoid disclosing any information that could potentially reveal their assigned group to the evaluators.

#### Blinding.

In the event of any suspicion or potential unblinding, the implicated evaluator will be promptly replaced to ensure the integrity of the blinding process. At the end of the study, evaluators will guess which participants were part of each group in order to assess the success of the blinding procedure by estimating the accuracy rate of participant allocation identification (masking effectiveness assessment). This protocol is designed to maintain the objectivity and reliability of the outcome assessments.

### Data collection, management, and analysis

The data will be the responsibility of the department. Self-report measures will be sent to participants via REDCap, and additional clinical information will be obtained by the physician during consultations. The signed informed consent form, in physical format, will be securely stored within the department. Data will be collected only while the participant is enrolled in the study. In case of dropout, information regarding the reasons for discontinuation will also be collected.

#### Statistical methods.

Data analysis will be conducted by a researcher blinded to the treatment conditions. A descriptive analysis of sociodemographic data, the impact of psychoeducation, and symptom severity will be conducted. Depending on the normality of our data, results will be addressed by parametric or non-parametric analysis. We expect to use variance and multivariate analysis, correlations, and mixed models.

The dependent variable (DV) will be the change in BDI-II scores from baseline to post-intervention. The independent variable (IV) will be the treatment group (PE). All analyses will be performed using the STATA BE [37] 18 or RStudio software [38], and data will be considered statistically significant when *p* is equal to or less than 0.05. We will consider the intention-to-treat analysis and data imputation of participants who have at least 10% of the data. Intention-to-treat (ITT) analysis will be conducted, and missing data will be imputed using multiple imputation for participants with at least 10% of their data collected. Subgroup analyses, sensitivity analyses, effect size calculations, and the Number Needed to Treat (NNT) will be performed as necessary.

#### Cost-effectiveness analysis of the intervention.

Aiming to understand the relevance of the program cost- cost-effectiveness of the intervention will also be examined. Data will be retrieved from the treatment program, considering the costs involved with staff, dose of antidepressants, time of response, and relapse. Scores from secondary outcomes will also be considered in our final cost-effectiveness analysis. This analysis will determine the incremental cost-effectiveness ratio (ICER), comparing the intervention PE to the treatment-as-usual (TAU) control group. For this aim, we will consider personnel costs, mediation costs, program implementation costs, and resource utilization.

### Monitoring

#### Data monitoring.

Data monitoring will be conducted by the study manager, who will have access to all records in order to identify any data entry issues or factors that may lead to participant discontinuation. Additionally, one of the senior research coordinators will also have access to the data to ensure quality control and certify protocol compliance.

*Interim analysis:* This study protocol incorporates a planned interim analysis to assess the preliminary efficacy of the treatment and to monitor rates of recruitment and retention. The analysis will be conducted after 50% of participants have been enrolled and completed the treatment. Results from BDI-II, as an increase in knowledge, will serve as the primary objective for interim analysis. Our study may be modified or prematurely terminated if interim analysis reveals clear evidence of treatment efficacy (less than 50% of change in BDI II)/futility (less than 5 points in BDI II scale) or insufficient participant recruitment or retention rates significantly impacting the study feasibility (less than 70% of retention). Analysis will be performed using O’Brien-Fleming, with an adjusted alpha value of approximately 0.0001 to 0.001. An independent committee will review the data, and in case of termination, participants will be properly informed and assisted. Results from the interim analysis will be documented and published.

#### Harms.

This study involves minimal risk. However, mild emotional reactions may occur in response to the topics addressed during the educational sessions or while completing self-report questionnaires. Additionally, any adverse effects related to the use of prescribed medications will be monitored. In all cases, the physicians responsible for each participant will assess the situation and take appropriate clinical action, by considering medical and ethical standards. All adverse events, whether solicited or spontaneously reported, will be recorded by the evaluator and closely monitored to ensure participant safety throughout the study.

#### Auditing.

The research team will meet at the end of each 6-week cycle of the active group to review the study procedures. In addition, quarterly meetings will be held to evaluate the study as a whole.

### Ethics and dissemination

#### Research ethics approval.

This research has been performed in accordance with the Declaration of Helsinki. Approval to conduct the study was received from the Faculty of Medicine of the University of São Paulo – FMUSP (76212623.1.0000.0068). The study design has been developed in accordance with CONSORT guidelines [39].

#### Protocol amendments.

If any protocol modifications are required, they will be discussed within the research group. Senior researchers A.M.C. and R.A.M. will make the final decision, and all changes will be documented in the registration system and updated in the protocol.

#### Consent or assent.

Written informed consent will be obtained from all participants, and only after reading and providing consent for participation will participants be randomly assigned to one of the main groups. After completing the six-month phase, patients will undergo a crossover. No washout will be made. All participants will receive a copy of the consent form.

#### Confidentiality.

Access to the collected data will be restricted to the researchers directly responsible for the study. Data will be stored securely on the REDCap platform, while physical documents, such as signed consent, will remain under the responsibility of the department and stored in a secure location. All procedures are designed to ensure the confidentiality and protection of participant information. The information obtained will be analyzed together with other individuals, and no personal identification will be disclosed.

### Ancillary and post-trial care

This study does not involve any biological interventions or procedures that could lead to physical harm. Therefore, no specific provisions for ancillary or post-trial biological care are required. As the nature of the study presents minimal risk, no compensation for harm is anticipated. However, should any unforeseen issues arise related to participation, appropriate measures will be taken by the research team in accordance with institutional policies and ethical guidelines.

### Dissemination policy

We expect to share our findings at scientific conferences and congresses; also, we wish to publish all steps in scientific journals. Furthermore, if results are positive, we expect to implement the PEG-D program in public systems.

## Discussion

Depression represents a serious, prevalent, and persistent illness, posing a significant challenge in the field of mental health [1]. Standardizing and testing non-pharmacological treatments, such as psychoeducation, may be an important and imperative need to effectively address these conditions and minimize the adverse impact on patients and their families [2–41].

The proposed treatment (PEG-D) was developed to provide psychoeducation and training to participants regarding depression, covering clinical characteristics, causes, treatment, lifestyle, and practical strategies [42]. Confronting the inherent challenges of the disorder, we expect that participants receiving the intervention will gain greater insight and training about depression. We anticipate that this empowerment will have an effect on the level of depression, on adherence, quality of life, and cognitive and behavioral measures compared to the control group [43,44].

In this context, our study stands out as the first randomized controlled clinical trial to adopt knowledge level as the primary outcome in a psychoeducational intervention for depression. This approach reflects a conceptual shift: rather than focusing exclusively on symptom reduction, we propose that increased knowledge—and the resulting changes in patients’ relationship with the illness and its treatment—may represent a central mechanism through which psychoeducation exerts its effects. Therefore, it is essential to consider knowledge as an outcome measure.

Potential positive results will contribute to the advancement of clinical and psychosocial approaches in managing depression, presenting itself as an attractive and economically viable alternative to adjunctive treatment. Additionally, there is the potential for replicating the program in other formats and modalities, primarily aiming to improve the well-being of those affected.

### Trial status

This trial is currently ongoing and open for participant recruitment, having commenced in March 2024. The study protocol was registered on clinicaltrials.gov in June 2024. Final assessments for all participants are anticipated to be completed by the end of December 2027.

## Supporting information

S1 Fig SPIRIT checklist. Completed checklist following the SPIRIT guidelines, detailing the items that comprise the study protocol.
(DOCX)

S1 File Study protocol (Portuguese version). Full version of the study protocol written in Portuguese, including methodological information and planned procedures.
(DOCX)

S2 File Study protocol (English version). Full version of the study protocol translated into English, maintaining fidelity to the original Portuguese content.
(DOCX)

## Abbreviations

BDI: Beck Depression Inventory
CBAS: Cognitive Behavioral Avoidance Scale
CGI: Clinical Global Impression Scale
DSM: Diagnostic and Statistical Manual of Mental Disorders
DTS: Depressive Thoughts Scale
GAF: Global Functioning Rating Scale
HAM-D: Hamilton Rating Scale for Depression
MDD: major depressive disorder
MADRS: Montgomery-Asberg Depression Rating Scale
PE: psychoeducation
PEG-D: Psychoeducation Group for Depression
SCID: structured clinical interview
TAU: usual treatment
UKU: Udvalg for Kliniske Undersøgelser
YMRS: Young Mania Rating Scale
