# Supplementary material for: Psychoeducation Group for Depression (PEG-D): Study protocol for a prospective, randomized, single-blind, crossover trial
Source: PLoS One. 2025 Aug 8;20(8):e0329006. doi: 10.1371/journal.pone.0329006 (PMC12334054; doi:10.1371/journal.pone.0329006)
Supplement: S1 File — Full version of the study protocol written in Portuguese, including methodological information and planned procedures. (DOCX) [file pone.0329006.s002.docx]

**Psicoeducação para transtorno depressivo maior: desenvolvimento e avaliação de eficácia**

**Pesquisador executante**: Dra. Adriana Munhoz Carneiro; Sara Lisboa Teodoro da Silva, Pedro Henrique Nunes Souto, Jainan Rodrigues Barretto, Lilian Hupfeld Moreno, Fernando Cordeiro Pimentel, Fernando dos Santos Fernandes

**Pesquisador Responsável**: Ricardo Alberto Moreno

**2023**

**Resumo**

A depressão maior é um transtorno complexo, considerado como uma doença altamente incapacitante e uma das mais prevalentes em todo o mundo e que frequentemente requer uma combinação de intervenções farmacológicas e psicoterapêuticas. A psicoeducação surgiu como uma intervenção psicossocial promissora que pode aumentar a eficácia dos tratamentos farmacológicos e a adesão ao tratamento de um modo geral, sendo caracterizada por intervenções psicoterapêuticas e educacionais. O objetivo do presente estudo é de testar a eficácia de um programa psicoeducativo quanto a melhora dos sintomas e aspectos cognitivos comportamentais de pacientes diagnosticados com Transtorno Depressivo Maior. Para isso, espera-se que sejam recrutados 338 sujeitos, que deverão ser divididos entre grupo ativo (no caso, que receberá a psicoeducação) e grupo controle (que receberá o tratamento usual – no caso, apenas o segmento ambulatorial). Espera-se que a psicoeducação possa contribuir para a melhora dos sintomas e aspectos cognitivos comportamentais, como redução de esquiva das relações e melhora de seus padrões de pensamento.

**Palavras chave:** psicoeducação, intervenção psicoeducacional, depressão, transtorno depressivo maior.

**Sumário**

[**1. Introdução…………………………………………………………………………………………**](#_heading=h.gjdgxs) **4**

[**2. Objetivos…………………………………………………………………………………………..**](#_heading=h.30j0zll) **7**

[**3. Justificativas………………………………………………………………………………………**](#_heading=h.1fob9te) 7

[**4. Métodos……………………………………………………………………………………………**](#_heading=h.3znysh7) **8**

**4.1 Participantes……………………………………………………………………….………….8**

**4.2 Elegibilidade……………………………………………………………………….………….8**

**4.3 Instrumentos de Avaliação……………………………………………………….………..9**

**4.4 Procedimento………………………………………………………………………………..13**

**5. Plano de Trabalho e Cronograma…………………………………………………………....15**

**6. Plano de Análise de Dados…………………………………………………….…………..….16**

**7.Cronograma ......................................................................................................................18**

**Referências………………………...……………………………………………..…………….......19**

1. **Introdução**

O Transtorno Depressivo Maior (TDM) se caracteriza pela apresentação clínica de um grupo de sintomas por um período mínimo de duas semanas. É composto por sintomas cardinais, sendo esses, humor deprimido e/ou perda de interesse, somados a sintomas de alterações no peso, sono, psicomotricidade, energia, percepção e cognição (APA, 2022; CARNEIRO, 2019). É considerado uma doença altamente incapacitante e uma das mais prevalentes em todo o mundo (WHO, 2016).

Guitérrez-Rojas et al. (2020) publicaram uma recente revisão sistemática reunindo evidências sobre a epidemiologia do TDM ao redor do mundo corroborando com a alta prevalência em diversos países, mesmo em continentes e culturas diferentes. É visto ainda uma prevalência maior em países ocidentais, entretanto deve-se levar em consideração que a manifestação clínica da doença varia de acordo com diferenças culturais, acarretando em divergências nos critérios diagnósticos adequados para esses casos (GUTIÉRREZ-ROJAS et al., 2020).

Além de prejuízos funcionais, observou-se a associação entre TDM e outros quadros psiquiátricos, físicos e de saúde, de modo que o paciente com o diagnóstico pode apresentar no curso da doença um transtorno de ansiedade, assim como a ansiedade pode abrir caminho para a depressão (TALKOVSKY et al., 2017; MOFFITT et al., 2007; BEESDO et al., 2007). À medida que se experimenta um episódio, as probabilidades de ocorrer um novo aumentam progressivamente (BOCKTING et al., 2015), sendo que mesmo que recebam tratamento, ao menos 80% dos indivíduos apresentarão um segundo episódio. (FLECK et al., 2009).

Em relação ao tratamento, a farmacoterapia é indicada como primeira opção, porém, debate-se sua eficácia em prevenir recaídas e novos episódios (CARNEIRO; DOBSON, 2016). Algumas das explicações propostas à cronicidade e consequentes altas taxas de recaída e recorrência do quadro depressivo giram em torno da vulnerabilidade individual à doença e das "cicatrizes" deixadas por um ou mais episódios (BOCKTING et al., 2015). De 30 a 50% dos pacientes tendem a não apresentar a resposta desejada ao tratamento farmacológico (ROZENTHAL et al., 2004). Contudo, da porção que responde positivamente ao tratamento, até 60% podem não apresentar remissão dos sintomas com a primeira intervenção medicamentosa, e 50% a alcançam em um segundo momento com a troca do medicamento ou então combinando tratamentos (DEL PORTO et al., 2009). Assim, diante desse contexto vivenciado pelos clínicos durante o tratamento se torna visível o curso crônico e recorrente experimentado pelos pacientes. Uma vez que a reincidência de um episódio depressivo se mostra mais a regra do que a exceção, desenvolver estratégias para além da farmacologia que complementam o tratamento pode se mostrar benéfico.

Srinivasan, Cohen e Parikh (2003), avaliando a perspectiva do paciente sobre a etiologia da depressão, descreveram que os pacientes não apoiam a explicação biomédica dominante de que alterações biológicas são centrais na depressão. Os pacientes também rejeitaram fortemente atribuir a depressão a déficits espirituais ou a um estilo de vida pouco saudável, assim sugerem que a visão dos pacientes com depressão são frequentemente não biomédicos. Os autores ainda descrevem que essa visão por parte dos pacientes relaciona-se às implicações clínicas, uma vez que a percepção das causas da doença mental é um importante determinante da adesão ao tratamento e que as crenças e atitudes dos pacientes devem ser incorporadas na psicoeducação.

A psicoeducação é um tratamento adjuvante que tem emergido como uma intervenção eficaz que visa melhorar o nível de compreensão dos pacientes, familiares e cuidadores sobre a doença, possibilitando a participação dos mesmos no tratamento. (Lukens & McFarlane, 2004) A psicoeducação é uma parte padrão do tratamento para quase todos os tipos de transtornos mentais e é considerada uma forma de intervenção psicoterapêutica básica e adicional para pacientes, podendo ser aplicada em diferentes fases do tratamento (ajudando no diagnóstico inicial, durante a fase de tratamento, recuperação e orientação familiar). Assim, o objetivo central da psicoeducação é oferecer educação e estratégias terapêuticas para melhorar a qualidade de vida, fornecendo conhecimento sobre várias facetas de sinais de doença, sintomas, curso, e prognóstico, dissipando equivocos e inconsciência diminuindo a possibilidade de recaídas. (Bhattacharjee *et al*., 2011)

Sobre a aplicabilidade da psicoeducação no TDM, em uma revisão sistemática (Tursi *et al*., 2013) que avaliaram a eficácia da PE em pacientes adultos com depressão, é relatado que embora as publicações nessa área ainda sejam limitadas, os artigos selecionados sugerem que a psicoeducação é eficaz para melhorar o curso clínico, a adesão ao tratamento e o funcionamento psicossocial dos pacientes. Porém os autores também relatam que os estudos foram conduzidos com formas de intervenção muito diferentes, fazendo com que a comparação fosse ainda mais difícil, pois não foram bem desenhados ou bem descritos no que diz respeito ao tratamento, abordagens terapêuticas que empregavam.

Assim, ainda não há diretrizes ou protocolos bem estabelecidos de como deve ser conduzida a psicoeducação no TDM, o que justifica a necessidade de novos estudos que contribuam para explorar as evidências de sua eficácia como técnica auxiliar no tratamento.

1. **Objetivos**
2. Avaliar a eficácia de um programa psicoeducativo na melhora dos sintomas depressivos;
3. Investigar se os pensamentos disfuncionais e comportamentos de evitação modificam-se após intervenções psicoeducativas;
4. Observar o impacto na aderência ao tratamento dos pacientes que realizam psicoeducação, comparado aos pacientes que não realizaram a prática psicoeducativa.

**3. Justificativas**

- falta de trabalhos padronizados sobre psicoeducação no TDM;
- incidência e custos envolvidos ao tratamento do transtorno depressivo maior;
- dificuldades em aderência ao tratamento dos TDM.

1. **Métodos**

**4.1 Desenho**

O estudo será um ensaio clínico randomizado, prospectivo, cruzado e de local único, no qual a intervenção psicossocial (Psicoeducação) será oferecida combinada com o tratamento usual. O grupo controle que iniciar apenas o tratamento usual, após o seguimento de seis meses também poderá receber a intervenção psicoeducacional.

**4.2 Participantes**

338 pacientes com diagnóstico de depressão maior unipolar diagnosticado por psiquiatra por meio da SCID, devendo estes serem recrutados para pesquisa no Instituto de Psiquiatria (IPq) do HCFMUSP, não tendo restrição de sexos.

**Tamanho da amostra**

Um cálculo do tamanho da amostra foi conduzido para uma MANOVA de medidas repetidas com um fator entre sujeitos, considerando um tamanho de efeito moderado (f = 0,25), nível de significância de 0,05, poder estatístico de 0,95, dois grupos, cinco medidas repetidas e uma correlação entre medidas repetidas de 0,70. O teste F foi usado com o algoritmo de O'Brien-Shieh e estatística de rastreamento de Pillai (V de Pillai = 0,0759878), resultando em um tamanho de amostra total necessário de 160 participantes, com um parâmetro de não centralidade (λ) de 13,16, valor crítico de F de 3,90, 1 grau de liberdade do numerador e 158 graus de liberdade do denominador, garantindo poder suficiente (0,9500442) para detectar o efeito proposto. Para contabilizar a potencial perda de participantes, aumentamos para esse número 20% da taxa de perda, para garantir poder suficiente para o estudo, apesar de potenciais desistências, resultando em um tamanho total de amostra de 192 participantes.

**4.3 Elegibilidade**

**Critérios de inclusão:**

- Pacientes de ambos os sexos;
- Idades entre 18 e 65 anos;
- Diagnóstico de TDM feito por um clínico de acordo com os critérios do DSM-V-TR,
- Os pacientes deverão ter gravidade de moderada a grave segundo a HAMD (escores entre 14 a 23);
- Termo de consentimento esclarecido e assinado.

**Critérios de exclusão:**

- Ter idade diferente da indicada no critério de inclusão;
- Ter doenças clínicas ou neurológicas graves instáveis;
- Pacientes com depressão pós-parto ou outros tipos de transtornos depressivos (transtorno disruptivo de desregulação do humor, transtorno disfórico pré-menstrual, transtorno depressivo induzido por substância/medicamento, transtorno depressivo devido a outra condição médica, outro transtorno depressivo especificado e transtorno depressivo não especificado);
- Outros transtornos psiquiátricos;
- Ideação suicida (pontuação >2 no item 3 do HAMD);
- Pacientes com sintomas psicóticos ativos;
- Pacientes já fazendo algum outro tratamento para TDM que não seja possível realizar washout no caso de medicação.

**Critérios para descontinuação**

- Mais de duas sessões ao longo das 10 semanas, seja tratamento médico ou psicoeducação.
- Aqueles que após de oito semanas em uso de Sertralina não apresentarem respostas (<25%).
- Presença de sintomas hipomaníacos em qualquer momento do tratamento (Young <7)

**4.4 Instrumentos de avaliação:**

*Fase seleção:*

Entrevista Estruturada para o DSM IV– Transtornos eixo I – Versão Clínica (SCID-CV): desenvolvida com o intuito de padronização dos procedimentos diagnósticos psiquiátricos, por meio de entrevista. Neste trabalho, utilizar-se-á a versão traduzida para o português (Del-Ben & cols., 2001) composta por 15 perguntas, a partir das quais são preenchidos os pré-requisitos do manual DSM-IV para o diagnóstico do transtorno. O estudo de confiabilidade foi realizado por Del-Ben e colaboradores (2001), em pacientes psiquiátricos de um hospital do interior de São Paulo. Foi empregada a metodologia de teste-reteste, com intervalo de dois dias entre as entrevistas realizadas. Participaram 45 pacientes, com idade média de 34,9 anos (DP=11,8), a maioria mulheres (60%). O índice de concordância para o diagnóstico (Kappa) foi maior que 0,90, com significância a 1%, o que levou à conclusão de que a escala possui boa confiabilidade mesmo não apresentando todos os critérios que a versão original para pesquisa. Este instrumento será aplicado apenas no grupo de depressivos.

Escala de Depressão de Hamilton: escala multidimensional de heteroavaliação, considerada como “padrão ouro” para a avaliar a gravidade do episódio depressivo em pacientes portadores de transtornos do humor, entretanto, não se constitui como instrumento diagnóstico para identificação de depressão. A versão traduzida para o Brasil do questionário de avaliação foi realizada por Carvalho, Lima, Azevedo e Caetano (1993), os quais realizaram o estudo de validação aplicando aversão retro- traduzida em 63 estudantes universitários bilíngues. A HAM-D possui três versões, sendo elas de 17, 21 ou 24 itens, com respostas pontuadas de 0 a 4, sendo no presente estudo aplicada a de 24 itens. A avaliação do humor depressivo considera a presença/ausência dos sintomas conforme a última semana. Possui em sua maior parte sintomas cognitivos e vegetativos, avaliando em menor número de itens de fatores sociais, motores, ansiedade e humor, e permite a classificação da depressão leve, moderada ou grave. A confiabilidade da escala HAM-D é citada apenas em sua versão internacional, que varia de 0,83 a 0,94.

*Fase segmento:*

Questionário de Identificação: questionário utilizado com objetivo de coletar informações de caracterização da amostra para a pesquisa, como sexo, idade, estado civil, histórico de diagnóstico familiar e se a pessoa já está passando por tratamento para a depressão.

Questionário de conhecimento: questionário desenvolvido pelos autores a partir de informações que serão discutidas em cada semana do grupo psicoeducacional, com o objetivo de avaliar o conhecimento sobre aspectos envolvidos na depressão bem como aprendizagem e generalização do conteúdo pelo participante, contendo itens com respostas de múltipla escolhas e abertas.

Cognitive Behavioral Avoidance Scale –CBAS: escala elaborada por Ottenbreit e Dobson (2004) a partir dos pressupostos de Fester (1973) que postula a importância de se observar as relações de evitação para provocar a modelagem e posterior mudança de comportamento. A escala foi desenvolvida especificamente com frases referentes a estilos de evitação típicos de deprimidos e possui 31 itens, divididos em quatro fatores, quais sejam, Comportamental/social (8 itens), referentes a evitar sair ou realizar atividades, evitar eventos sociais; Cognitivo não social (10 fatores) referindo se a pensamentos de falhas, derrotas e de deixar as possibilidades passarem, Cognitivo Social (7 itens) com itens sobre problemas em relacionamentos sociais relacionados a incapacidade de mudar a forma de reforçamentos provenientes dessa fonte e Comportamental não social (6 itens) em evitar atividades novas, não se sentir hábil para realizar tarefas e acreditar grande parte que tudo o que faz terá como consequência falhas. A confiabilidade da versão canadense dos fatores variou de 0.75 a 0.80. Para o estudo brasileiro, a autora do presente projeto entrou em contato com o autor, que disponibilizou a versão em inglês para o processo de tradução e retro tradução. Para o processo de tradução, foram selecionadas duas psicólogas com conhecimento avançado em inglês, uma delas que trabalha diretamente com transtornos de humor e em ambulatório de Transtorno Depressivo e Bipolar e outra sem contato com esse tipo de população (neuropsicologa). Foi entregue a CBAS e solicitado para que traduzissem da forma que melhor entendessem. Após entregue as traduções, a autora condensou o conteúdo das traduções e enviou uma versão em português para uma brasileira residente há mais de 6 anos nos Estados Unidos para retrotraduzir a versão portuguesa para o inglês. A partir do resultado, foi conversado com o autor da escala e comparado o resultado das traduções. A versão brasileira assim, terá o mesmo número de itens e apenas um item foi modificado, de forma a condizer melhor com a realidade cultural.

Escala de Pensamentos Depressivos: instrumento construído por meio da elaboração de itens de acordo com a tríade cognitiva de Beck (Beck, Rush, Shall & Emery, 1982) e em outros instrumentos de avaliação de crenças que se encontrassem entre os mais utilizados nos artigos científicos e/ou que tivessem em seu conteúdo itens que avaliassem pensamentos relacionados à depressão com base na tríade (Instrumento de Crandell sobre a tríade cognitiva; Questionário de pensamentos automáticos; Questionário de estilo atribucional; Questionário de estilo atribucional ampliado; Escala de crenças; Inventário da tríade cognitiva; Irrational values scale –IVS; Mood Survey; Self rating depression scale SDS; Children cognitive assessment questionnaire –CCAQ; Dysfunctional attitude scale -DAS). A partir da elaboração de itens pelos autores do projeto e da tradução dos itens contidos em outras escalas que avaliassem depressão, foram mantidos aqueles que diziam respeito às crenças de incapacidade, relacionadas por A. T. Beck (1982) como aquelas referentes a sentimentos de autorecriminação, culpa, sentimento de inutilidade, baixa estima frente às questões externas (relação do sujeito com os outros e o mundo). A versão final do instrumento possui 26 itens, divididos em dois fatores, sendo o primeiro baixa auto estima/desesperança, com 16 itens referentes a percepção negativista do sujeito quanto a si e as suas perspectivas de futuro e o segundo sobre funcionalidade nas relações, com 10 itens sobre a avaliação positiva que o sujeito apresenta sobre sua relação com os outros e como avalia o suporte que recebe. A escala possui confiabilidade de 0.93, sendo 0.93 o Fator 1 e 0.89 o Fator 2. Para a contagem, quanto maior a pontuação, maiores os pensamentos disfuncionais.

Beck Depression Inventory second edition –BDI II (Goreinstein, Pang, Argimon & Werlang, 2011): validado para o Brasil, é composto por 21 itens e mensura a intensidade da depressão tanto em pacientes diagnosticados quanto em casos prováveis na população normal entre uma faixa etária de 13 e 59 anos. É uma escala tipo *Likert* de quatro pontos, em que quanto maior a pontuação, maior o nível de depressão nos últimos 14 dias. Este instrumento possui algumas diferenças da primeira versão, como por exemplo, o item que avalia perda de apetite, insônia e retraimento social, que foram reelaborados conforme a 4ª edição do DSM IV (APA, 1994). Quatro itens foram retirados da primeira versão (perda de peso, mudança na auto-imagem, percepções somáticas e dificuldades de trabalhar) e substituídos por agitação, desvalorização, dificuldade de concentração e falta de energia. O instrumento possui 21 itens, sendo que quanto maior a pontuação, maior a intensidade da depressão.

Generalized Anxiety Disorder 7 - GAD 7 (Spitzer, Kroenke, Williams, & Löwe, 2006): Consiste em um instrumento breve e de autorrelato, medindo a frequência de sintomas ansiosos nas últimas duas semanas. Desenvolvida com sete itens e com uma pontuação de 0 a 3, obtém-se o escore total a partir da soma dos itens, variando entre 0 e 21 pontos. Os participantes são classificados em grupos de acordo com seu nível de ansiedade, 0-4 como mínimo, 5-9 leve, 10-14 moderado e 15-21 severo. Na versão em Português, a escala demonstrou uma boa fi dedignidade; sendo que tanto o coeficiente de Cronbach alfa (α = 0,916) e rho coeficiente de confi abilidade composta (ρ = 0,909) foram adequados. (Moreno et al., 2016).

Escala De Avaliação Global De Funcionamento - GAF: O GAF é uma escala de avaliação de funcionamento que varia de 0 a 100, sendo as pontuações mais altas indicativo de níveis mais altos de funcionamento.

Clinical Global Impression Scale - CGI (Busner & Targum, 2007): A CGI é uma medida resumida geral determinada pelo médico que leva em consideração todas as informações disponíveis, incluindo o conhecimento da história do paciente e o impacto dos sintomas na capacidade funcional do paciente. Compreende dois componentes, o Clinical Global Impression – Severity Scale (CGI-S), que avalia a gravidade da doença, com escores variando de 1 (normal, não doente) a 7 (doença mental extremamente grave) e a Clinical Global Impression – Improvement Scale (CGI-I), que avalia a melhora global desde o início do tratamento, com escores variando de 1 (muito melhor) a 7 (muito pior).

Behavioral Activation for Depression Scale Short Form (BADS-SF) (Manos et al., 2011) : A BADS é uma escala desenvolvida a partir do modelo comportamental da depressão. Tem como objetivo mensurar aspectos comportamentais que estão correlacionados à mudanças clínicas. Possui duas versões, uma estendida e outra em um formato breve com 25 e 9 itens, respectivamente.Cada item é mensurado em escala de 0 a 6 pontos, divididos em dois fatores: ativação e esquiva. Será utilizada no estudo a BADS-SF, de 9 itens, validada para a população brasileira. (Aschar et al. 2021).

ShapsBr-Snaith-Hamilton Pleasure Scale (Sanith et. al, 1995): Escala de Prazer SHAPS é um instrumento utilizado para avaliar a experiência de prazer ou a antecipação de uma experiência prazerosa. Esta escala consiste em 14 itens, nos quais os participantes indicam seu nível de concordância em uma escala de quatro pontos: "concordo definitivamente", "concordo", "discordo", e "discordo definitivamente" Uma pontuação mais alta indica um maior nível de anedonia. Validade na versão em portugues por Jesus-Nunes et. al ( 2021), apresentou consistência interna do instrumento, avaliada pelo coeficiente alfa de Cronbach, demonstrou ser adequada (α = 0,759; n = 228; 14 itens).

Young Mania Rating Scale (YMRS) (Young et al., 1978): escala composta por 11 items, pontuados de 0 a 4 e de 0 a (irmabilidade, fala, conteúdo do pensamento e comportamento disruptivo-agressivo, pontuados em dobro para compensar a ausência de cooperação do paciente). E a escala mais atilizada em estudos clinicos com pacientes maniacos, com elevado indice de confiabilidade entre avuliandures (em tomo de 0,93 pata a pontuação total e entre 0.66 * 0.92 para os itens individuais) Para a Young, foi adotada a versão tradunda por Vilela (2000), com ponto de corte <7 come indicativo de sintomas significativos de mania/hipomania.

**4.4 Procedimentos**

Este estudo será realizado de forma longitudinal visando investigar o impacto de um programa de psicoeducação para a depressão. Para tal, os participantes pacientes psiquiátricos devem preencher os critérios de inclusão, que necessariamente indiquem presença de Episódio/Transtorno depressivo maior ou Transtorno Depressivo Recorrente sem sintomas psicóticos. Os pacientes serão alocados em um dos dois grupos (controle ou experimental) mediante randomização simples (1:1) que será realizada por meio do site [www.sealedenvelope.com](http://www.sealedenvelope.com) . Este site gera blocos de tamanhos e comprimentos aleatórios.

O estudo será realizado por meio dos passos já descritos acima, entretanto, vale reafirmá-los. A entrada de pacientes para a pesquisa será feita após cumpridos os critérios de inclusão, aprovação do projeto pelo comitê de ética da instituição e consentimento dos sujeitos. A testagem será feita com instrumentos para verificação de gravidade de sintomas de depressão (HAM-D), pensamento (EPD), comportamentos de evitação (CBAS e BADS) níveis/intensidade de depressão (BDI II) e um questionário de identificação. Para entender melhor sobre o impacto da abordagem psicoeducativa, buscou-se inserir outros instrumentos que facilitassem esta interpretação, como a CGI e GAF para impactos em qualidade de vida, ansiedade (GAD-7), e experiencia de prazer (Shaps). A duração da aplicação dos instrumentos dependerá das fases, sendo o tempo estipulado para a aplicação do grupo clínico 1 hora e 40 minutos, contando com a SCID. Para a psicoeducação, o tempo dos encontros deverá ser de 90 minutos, ocorrendo semanalmente ao longo de seis semanas (**ver Tabela 1**).

**Tratamento usual (TAU):** O tratamento será iniciado com Sertralina (ISRS) – doses de 50 mg e ajustes conforme necessário de acordo com o perfil do paciente. Os ajustes de dosagem serão feitos de acordo com as pontuações na escala de avaliação de efeitos colaterais HAMD e Udvalg for Kliniske Undersøgelser (UKU) (Lingjaerde et al., 1987).

Após 8 semanas, se o paciente apresentar remissão, continuará em monoterapia com sertralina. Em caso de recaída, Bupropiona, Quetiapina, Lítio ou Aripiprazol podem ser complementares. Neste caso, os medicamentos complementares serão determinados de acordo com o perfil do paciente, pelo médico. Se houver resposta acima de 25% dos sintomas, mas sem remissão, o paciente procederá à potencialização com as medicações citadas e se não houver melhora de pelo menos 25%, o paciente será considerado desistente. Associações com Lorazepam, se necessário, serão permitidas em qualquer fase do estudo.

**Esquema de aplicação do programa de psicoeducação:** O formato das sessões será configurado, inicialmente, com cerca de 10 minutos de acolhimento (em que serão discutidos temas livres). Os 40 minutos seguintes serão destinados à exposição dos temas propostos, em esquemas de aulas interativas utilizando slides e será entregue uma versão impressa do mesmo material. Na sequência, 20 minutos serão reservados para discussão e treinamento sobre as informações oferecidas e os 20 minutos restantes, para revisão do que foi discutido e exposto, bem como para dúvidas e esclarecimentos. As sessões serão realizadas de forma presencial e aberta, permitindo que novos membros possam iniciar em qualquer uma das sessões. Aos participantes do grupo controle que optarem por receberem a psicoeducação, a estrutura seguirá a mesma.

Abaixo, a descrição dos temas abordados em cada sessão.

**Tabela 1. Sessões de psicoeducação**

| **Sessão** | **Tópicos** |
| --- | --- |
| **1. O que é depressão** | - Explicação aos participantes sobre a natureza e características clínicas do transtorno depressivo; - Diferenciar tristeza de depressão; - Sinais e sintomas; |
| **2. Causas e tratamento** | - Causas (fatores biológicos, psicológicos e ambientais); - Apresentação sobre quais tratamentos são recomendados para depressão; - Tratamento farmacológico e outras formas de tratamento. |
| **3. Identificando fatores de risco e lidando com crises** | - Que fatores contribuem para que a depressão não melhore? - O que fazer durante uma crise, que estratégias podem ajudar. - Identificando sintomas depressivos e o que fazer quando um novo episódio é detectado? - Métodos de manejo após identificação de novo episódio. |
| **4. Benefícios de adotar um estilo de vida regular** | - O papel do estilo de vida na depressão; - Importância do ciclo circadiano e das técnicas de higiene do sono. |
| **5. Resolução de problemas** | - Estilos de comunicação – desenvolvendo assertividade - Tomando decisões. |
| **6. Estratégias práticas** | - Verificação dos fatos (estrátégia cognitiva); - Acumular emoções positivas (importância das atividades prazerosas); - Mindfulness (importância de estar focado no momento presente, estratégias para observar, descrever e participar). |

**5. Plano de Trabalho e Cronograma de sua execução**

**Tabela 2.** Esquema de aplicação das escalas ao longo do projeto

|  | **V1** | **V2** | **Pós 3 meses** | **Pós 6 meses** | **Pós 12 meses** |
| --- | --- | --- | --- | --- | --- |
| **SCID** |  |  |  |  |  |
| **HAMD** |  |  |  |  |  |
| **BADS** |  |  |  |  |  |
| **SHARPS** |  |  |  |  |  |
| **CGI** |  |  |  |  |  |
| **GAF** |  |  |  |  |  |
| **GAD-7** |  |  |  |  |  |
| **CBAS** |  |  |  |  |  |
| **BDI II** |  |  |  |  |  |
| **EPD** |  |  |  |  |  |
| **Young** |  |  |  |  |  |
| **Questionário identificação** |  |  |  |  |  |
| **Questionário conhecimentos** |  |  |  |  |  |

**Legenda:** BDI II - Escala Beck de Depressão, CBAS - Escala Cognitivo Comportamental de Evitação, EPD - Escala de Pensamentos Depressivos, GAD - Transtorno de Ansiedade Generalizada, GAF - Escala de Avaliação Global de Funcionamento, SCID - Entrevista estruturada para o DSM IV, SHARPS - ShapsBr-Snaith-Hamilton Pleasure Scale V1. Entrada, antes da intervenção; V2. Final da sexta sessão de psicoeducação

**6. Plano de Análise de Dados**

Inicialmente, será realizada uma análise descritiva sobre os dados sociodemográficos, impacto da psicoeducação e gravidade dos sintomas. Considerando que os dados permitirão considerar os dados como paramétricos, o desfecho principal (redução de gravidade de sintomas) será avaliado pelos testes de diferença de média t de student, ANOVA e MANOVA de amostras dependentes [pré e pós] e independentes [GC e GE]. Todas as análises serão realizadas pelo programa STATA BE 18, e serão considerados estatisticamente significativos os dados quando forem p igual ou maior que 0.05.

**7. Cronograma**

É estipulado que o projeto tenha uma duração aproximada de 48 meses. Abaixo, encontra-se o cronograma esperado para o projeto.

|  | **Ano 1** | **Ano 2** | **Ano 3** | **Ano 4** |
| --- | --- | --- | --- | --- |
| **Escrita de dados** |  |  |  |  |
| **Atualização de referências bibliográficas** |  |  |  |  |
| **Levantamento de dados** |  |  |  |  |
| **Análise estatística** |  |  |  |  |
| **Preparo do manuscrito** |  |  |  |  |
| **Publicação** |  |  |  |  |

**Referências**

American Psychiatric Association. Diagnostic and Statistical Manual of Mental Disorders, Fifth Edition (DSM-5-TR). Arlington: American Psychiatric Publishing; 2022.

Beesdo K, Bittner A, Pine DS, et al. Incidence of social anxiety disorder and the consistent risk for secondary depression in the first three decades of life. Arch Gen Psychiatry 2007; 64: 903–12.

Bhattacharjee, D., Rai, A. K., Singh, N. K., Kumar, P., Munda, S. K., & Das, B. (2011). Psycho-education: A measure to strengthen psychiatric treatment. *Delhi Psychiatric Journal*, *14*(1),33-39.

Bockting, C.L., et al., A lifetime approach to major depressive disorder: The contributions of psychological interventions in preventing relapse and recurrence, Clinical Psychology Review (2015).

Busner J, Targum SD. The clinical global impressions scale: applying a research tool in clinical practice. *Psychiatry (Edgmont)*. 2007;4(7):28-37.

Carneiro, A.M.. Pensamentos disfuncionais e comportamentos evitativos ao longo do episódio depressivo: um estudo longitudinal. 2019. 154 f. Tese (Doutorado) - Faculdade de Medicina, Universidade de São Paulo, São Paulo, 2019.

Carneiro, A. M.; Dobson, K. S. Cognitive-behavioral treatment for major depressive disorder: a narrative review. Revista Brasileira de Terapias Cognitivas, v. 12, n. 1, 2016.

Cheng C, Cheung MW, Montasem A; 44 Members of the International Network of Well-Being Studies. Explaining differences in subjective well-being across 33 nations using multilevel models: universal per- sonality, cultural relativity, and national income. J Pers. 2016;84:46-58.

Del Porto J.A, Sarin LM, Moriyama T.S. Depressões resistentes. In: Lacerda ALT de, Quarantini L de C, Miranda-Scippa AMA, Del Porto JA. Depressão: do neurônio ao funcionamento social. Porto Alegre: Artmed; 2009. p. 163-179.

Fleck MP, Berlim MT, Lafer B, et al. (2009) Review of the guidelines of the Brazilian Medical Association for the treatment of depression. Revista Brasileira de Psiquiatria 31: S7–17.

Gutiérrez-Rojas L, Porras-Segovia A, Dunne H, Andrade-González N, Cervilla JA. Prevalence and cor- relates of major depressive disorder: a systematic review. Braz J Psychiatry. 2020;42:657-672. http://dx.doi.org/10.1590/1516-4446- 2019-0650

Jesus-Nunes AP, Coroa JPBB, Argolo FC, et al. Psychometric properties the of Brazilian Portuguese version of Snaith-Hamilton Pleasure Scale (SHAPS). *Trends Psychiatry Psychother*. 2021;43(1):23-29. doi:10.47626/2237-6089-2019-0066

Kessler RC. Epidemiology of women and depression. J Affect Dis- ord. 2003;74:5-13.

Kessler, R.C., Berglund, P., Demler, O., Jin, R., Merikangas, K.R., & Walters, E.E. (2005). Life- time Prevalence and Age-of-Onset Distributions of DSM-IV Disorders in the National Comorbidity Survey Replication. Archives of General Psychiatry, 62(6), 593–602. http://dx.doi.org/10.1001/archpsyc.62.6.593.

Luppino FS, de Wit LM, Bouvy PF, et al. Overweight, obesity, and depression: a systematic review and meta-analysis of longitudinal studies. Arch Gen Psychiatry 2010; 67: 220–29.

Manos, Rachel C., et al. “The Behavioral Activation for Depression Scale–Short Form: Development and Validation.” Behavior Therapy, vol. 42, no. 4, Dec. 2011, pp. 726–739, https://doi.org/10.1016/j.beth.2011.04.004

Moffitt TE, Harrington H, Caspi A, et al. Depression and generalized anxiety disorder: cumulative and sequential comorbidity in a birth cohort followed prospectively to age 32 years. Arch Gen Psychiatry 2007; 64: 51–60.

Moreno, A. L., Sousa, D. A., Souza, A. M. F. L. P, Manfro, G. G., Salum, G. A., Koller, S. H., Osório, F. L., Crippa, J. A. de S. (2016). Factor structure, reliability, and item parameters of the Brazilian-portuguese version of the GAD-7 questionnaire. Temas em Psicologia, 24(1), 367–376. https://doi.org/10.9788/TP2016.1-25

Rozenthal M, Laks J, Engelhardt E. Aspectos neuropsicológicos da depressão. Revista de Psiquiatria do Rio Grande do Sul. 2004 26:204-12.

Spitzer RL, Kroenke K, Williams JB, Löwe B. A brief measure for assessing generalized anxiety disorder: the GAD-7. *Arch Intern Med*. 2006;166(10):1092-1097. doi:10.1001/archinte.166.10.1092

Srinivasan, J., Cohen, N. L., & Parikh, S. V. (2003). *Patient Attitudes regarding Causes of Depression: Implications for Psychoeducation. The Canadian Journal of Psychiatry, 48(7), 493–495.*

Talkovsky AM, Green KL, Osegueda A, Norton PJ. Secondary depression in transdiagnostic group cognitive behavioral therapy among individuals diagnosed with anxiety disorders. J Anxiety Disord. 2017;46:56-64.

Tursi-Braga, MFS. Eficácia da psicoeducação para pacientes com depressão unipolar (dissertação). Ribeirão Preto: Universidade de São Paulo, Faculdade de Medicina, 2014. 137f.

World Health Organization (WHO). (2016). Fact sheet depression. Recuperado de: <http://www.who.int/mediacentre/factsheets/fs369/en/>.

Young R, Biggs J, Ziegler V. Meyer D. A rating scale for mania reliability, validity and sensibility. Br J Psychiatry, 1978,133-429-35.
