# Supplementary material for: Psychoeducation Group for Depression (PEG-D): Study protocol for a prospective, randomized, single-blind, crossover trial
Source: PLoS One. 2025 Aug 8;20(8):e0329006. doi: 10.1371/journal.pone.0329006 (PMC12334054; doi:10.1371/journal.pone.0329006)
Supplement: S2 File — Full version of the study protocol translated into English, maintaining fidelity to the original Portuguese content. (DOCX) [file pone.0329006.s003.docx]

**Psychoeducation for major depressive disorder: development and evaluation of effectiveness**

**Executing researcher:** Dra. Adriana Munhoz Carneiro; Sara Lisboa Teodoro da Silva, Pedro Henrique Nunes Souto, Jainan Rodrigues Barretto, Lilian Hupfeld Moreno, Fernando Cordeiro Pimentel, Fernando dos Santos Fernandes

**Responsible Researcher** Ricardo Alberto Moreno

**2023**

**Abstract**

Major depression is a complex disorder, considered a highly disabling disease and one of the most prevalent worldwide, which often requires a combination of pharmacological and psychotherapeutic interventions. Psychoeducation has emerged as a promising psychosocial intervention that can increase the effectiveness of pharmacological treatments and overall treatment adherence, and is characterized by psychotherapeutic and educational interventions. The objective of this study is to test the effectiveness of a psychoeducational program in improving symptoms and cognitive-behavioral aspects of patients diagnosed with Major Depressive Disorder. To this end,192 subjects are expected to be recruited, who should be divided into an active group (in this case, who will receive psychoeducation) and a control group (who will receive usual treatment – ​​in this case, only the outpatient segment). It is expected that psychoeducation can contribute to improving symptoms and cognitive-behavioral aspects, such as reducing avoidance of relationships and improving thought patterns.

**Keywords: psychoeducation, psychoeducational intervention, depression, major depressive disorder.**

**Summary**

**1.Introduction**[**…………………………………………………………………………………………**](#_heading=h.gjdgxs) **4**

[**2. Objectives…………………………………………………………………………….…………..**](#_heading=h.30j0zll) **7**

[**3.**](#_heading=h.1fob9te)**Justifications**[**……………………………………………………………………………………………..…**](#_heading=h.1fob9te) **7**

[**4. Methods……………………………………………………………………………………………**](#_heading=h.3znysh7) **8**

**4.1 Participants………….…………………………………………………………….………….8**

**4.2 Eligibility……………..…………………………………………………………….………….8**

**4.3 Assessment Instruments………………………………………………………….………..9**

**4.4 Procedure…..…………………………………………………………………………….…..13**

**5. Work Plan and Schedule………….……………………………………………..……….…....15**

**6. Data Analysis Plan………...…………………………………………………….…………..….16**

**7. Timeline…... ......................................................................................................................18**

**References………………………...……………………………………………..………….….......19**

1. **Introduction**

Major Depressive Disorder (MDD) is characterized by the clinical presentation of a group of symptoms for a minimum period of two weeks. It consists of cardinal symptoms, which are depressed mood and/or loss of interest, in addition to symptoms of changes in weight, sleep, psychomotor skills, energy, perception, and cognition (APA, 2022; CARNEIRO, 2019). It is considered a highly disabling disease and one of the most prevalent worldwide (WHO, 2016).

Guitérrez-Rojas et al. (2020) published a recent systematic review gathering evidence on the epidemiology of MDD around the world, corroborating its high prevalence in several countries, even on different continents and cultures. There is also a higher prevalence in Western countries, however, it should be taken into account that the clinical manifestation of the disease varies according to cultural differences, leading to divergences in the appropriate diagnostic criteria for these cases (GUTIÉRREZ-ROJAS et al., 2020). In addition to functional impairments, an association between MDD and other psychiatric, physical and health conditions has been observed, so that patients with the diagnosis may present an anxiety disorder during the course of the disease, just as anxiety can pave the way for depression (TALKOVSKY et al., 2017; MOFFITT et al., 2007; BEESDO et al., 2007). As an episode is experienced, the chances of a new one occurring progressively increase (BOCKTING et al., 2015), and even if they receive treatment, at least 80% of individuals will present a second episode (FLECK et al., 2009). Regarding treatment, pharmacotherapy is indicated as the first option, however, its effectiveness in preventing relapses and new episodes is debated (CARNEIRO; DOBSON, 2016). Some of the proposed explanations for the chronicity and consequent high rates of relapse and recurrence of depression revolve around individual vulnerability to the disease and the "scars" left by one or more episodes (BOCKTING et al., 2015). Between 30 and 50% of patients tend not to present the desired response to pharmacological treatment (ROZENTHAL et al., 2004). However, of the portion that responds positively to treatment, up to 60% may not present remission of symptoms with the first drug intervention, and 50% achieve it in a second moment with the change of medication or by combining treatments (DEL PORTO et al., 2009). Thus, in view of this context experienced by clinicians during treatment, the chronic and recurrent course experienced by patients becomes visible. Since recurrence of a depressive episode is more the rule than the exception, developing strategies beyond pharmacology that complement treatment may prove beneficial.

Srinivasan, Cohen, and Parikh (2003), assessing the patient's perspective on the etiology of depression, reported that patients do not support the dominant biomedical explanation that biological alterations are central to depression. Patients also strongly rejected attributing depression to spiritual deficits or an unhealthy lifestyle, thus suggesting that the view of patients with depression is often non-biomedical. The authors also describe that this view on the part of patients relates to clinical implications, since the perception of the causes of mental illness is an important determinant of adherence to treatment and that patients' beliefs and attitudes should be incorporated into psychoeducation.

Psychoeducation is an adjuvant treatment that has emerged as an effective intervention that aims to improve the level of understanding of patients, family members, and caregivers about the illness, enabling them to participate in treatment. (Lukens & McFarlane, 2004) Psychoeducation is a standard part of treatment for almost all types of mental disorders and is considered a form of basic and additional psychotherapeutic intervention for patients, and can be applied in different phases of treatment (helping in the initial diagnosis, during the treatment phase, recovery and family guidance). Thus, the central goal of psychoeducation is to offer education and therapeutic strategies to improve the quality of life, providing knowledge about various facets of disease signs, symptoms, course, and prognosis, dispelling misconceptions and unawareness, and decreasing the possibility of relapse. (Bhattacharjee et al., 2011)

Regarding the applicability of psychoeducation in MDD, in a systematic review (Tursi et al., 2013) that evaluated the effectiveness of PE in adult patients with depression, it is reported that although publications in this area are still limited, the selected articles suggest that psychoeducation is effective in improving the clinical course, adherence to treatment and psychosocial functioning of patients. However, the authors also report that the studies were conducted with very different forms of intervention, making the comparison even more difficult

1. **Objectives**
2. To evaluate the effectiveness of a psychoeducational program in improving depressive symptoms;
3. To investigate whether dysfunctional thoughts and avoidance behaviors change after psychoeducational interventions;
4. To observe the impact on treatment adherence of patients who undergo psychoeducation, compared to patients who did not undergo psychoeducational practice.

**3. Justifications**

- lack of standardized studies on psychoeducation in MDD;
- incidence and costs involved in the treatment of major depressive disorder;
- difficulties in adherence to MDD treatment.

**Methods**

**4.1 Design**

The study will be a randomized, prospective, crossover, single-site clinical trial in which psychosocial intervention (psychoeducation) will be offered in combination with usual care. The control group that starts only usual care will also receive the psychoeducational intervention after the six-month follow-up.

**4.2 Participants**

338 patients diagnosed with unipolar major depression diagnosed by a psychiatrist using the SCID, who should be recruited for research at the Institute of Psychiatry (IPq) of HCFMUSP, with no gender restrictions.

**Sample size:** The number of study participants was established through sample calculations using the G*Power software. A sample size calculation was conducted for a repeated measures MANOVA with a between-subjects factor, considering a moderate effect size (f = 0.25), significance level of 0.05, statistical power of 0.95, two groups, five repeated measurements, and a correlation among repeated measures of 0.70. The F test was used with the O'Brien-Shieh algorithm and Pillai’s Trace statistic (Pillai V = 0.0759878), resulting in a required total sample size of 160 participants, with a noncentrality parameter (λ) of 13.16, critical F value of 3.90, 1 numerator degree of freedom, and 158 denominator degrees of freedom, ensuring sufficient power (0.9500442) to detect the proposed effect. To account for potential participant attrition, we have increased to this number 20% of attrition rate, to ensure sufficient power for the study despite potential dropouts, resulting in a total sample size of 192 participants.

**4.3 Eligibility**

**Inclusion criteria:**

- Patients of both sexes;
- Age between 18 and 65 years;
- Diagnosis of MDD made by a clinician according to DSM-V-TR criteria;
- Patients must have moderate to severe severity according to HAMD (scores between 14 and 23);
- Signed informed consent form.

**Exclusion criteria:**

- Being of a different age than that indicated in the inclusion criteria;
- Having severe unstable clinical or neurological diseases;
- Patients with postpartum depression or other types of depressive disorders (disruptive mood dysregulation disorder, premenstrual dysphoric disorder, substance/medication-induced depressive disorder, depressive disorder due to another medical condition, other specified depressive disorder, and unspecified depressive disorder);
- Other psychiatric disorders;
- Suicidal ideation (score >2 in item 3 of the HAMD);
- Patients with active psychotic symptoms;
- Patients already undergoing some other treatment for MDD that cannot be washed out in the case of medication.

**Criteria for discontinuation**

- More than two sessions over the 10 weeks, whether medical treatment or psychoeducation.
- Those who do not respond after eight weeks of using Sertraline (<25%).
- Presence of hypomanic symptoms at any time during treatment (Young <7)

**4.4Assessment instruments:**

*Selection phase:*

Structured Interview for DSM IV – Axis I Disorders – Clinical Version (SCID-CV): developed with the aim of standardizing psychiatric diagnostic procedures through interviews. In this study, the version translated into Portuguese (Del-Ben et al., 2001) will be used, consisting of 15 questions, from which the prerequisites of the DSM-IV manual for the diagnosis of the disorder are met. The reliability study was carried out by Del-Ben et al. (2001), in psychiatric patients from a hospital in the interior of São Paulo. The test-retest methodology was used, with a two-day interval between interviews. A total of 45 patients participated, with a mean age of 34.9 years (SD=11.8), the majority of whom were women (60%). The agreement index for diagnosis (Kappa) was greater than 0.90, with significance at 1%, which led to the conclusion that the scale has good reliability even though it does not present all the criteria of the original research version. This instrument will be applied only to the group of depressives.

Hamilton Depression Scale: Hamilton Depression Scale: multidimensional hetero-evaluation scale, considered the “gold standard” for assessing the severity of depressive episodes in patients with mood disorders. However, it is not a diagnostic instrument for identifying depression. The Brazilian version of the evaluation questionnaire was developed by Carvalho, Lima, Azevedo and Caetano (1993), who conducted a validation study by applying the back-translated version to 63 bilingual university students. The HAM-D has three versions, with 17, 21 or 24 items, with responses scored from 0 to 4. In this study, the 24-item version was applied. The assessment of depressive mood considers the presence/absence of symptoms according to the last week. It mostly has cognitive and vegetative symptoms, evaluating a smaller number of items on social, motor, anxiety and mood factors, and allows the classification of mild, moderate or severe depression. The reliability of the HAM-D scale is cited only in its international version, which varies from 0.83 to 0.94.

*Phase segment:*

Identification Questionnaire: questionnaire used to collect sample characterization information for the research, such as sex, age, marital status, family diagnosis history and whether the person is already undergoing treatment for depression

Questionário de conhecimento: questionnaire developed by the authors based on information that will be discussed each week in the psychoeducational group, with the aim of assessing knowledge about aspects involved in depression as well as learning and generalization of the content by the participant, containing items with multiple choice and open answers.

Cognitive Behavioral Avoidance Scale – CBAS: scale developed by Ottenbreit and Dobson (2004) based on the assumptions of Fester (1973) who postulates the importance of observing avoidance relationships to provoke modeling and subsequent change in behavior. The scale was developed specifically with phrases referring to avoidance styles typical of depressed individuals and has 31 items, divided into four factors, namely, Behavioral/social (8 items), referring to avoiding going out or performing activities, avoiding social events; Non-social cognitive (10 factors) referring to thoughts of failure, defeat and letting possibilities pass by; Social cognitive (7 items) with items about problems in social relationships related to the inability to change the form of reinforcements coming from this source; and Non-social behavioral (6 items) in avoiding new activities, not feeling able to perform tasks and believing that everything one does will result in failure. The reliability of the Canadian version of the factors ranged from 0.75 to 0.80. For the Brazilian study, the author of this project contacted the author, who made the English version available for the translation and back-translation process. For the translation process, two psychologists with advanced knowledge of English were selected, one of whom works directly with mood disorders and in an outpatient clinic for Depressive and Bipolar Disorders, and the other who has no contact with this type of population (a neuropsychologist). The CBAS was given the translation and asked to translate it in the way they understood best. After delivering the translations, the author condensed the content of the translations and sent a Portuguese version to a Brazilian woman who had lived in the United States for over 6 years to back-translate the Portuguese version into English. Based on the results, the author of the scale was consulted and the results of the translations were compared. The Brazilian version will therefore have the same number of items and only one item was modified, in order to better match the cultural reality.

Depressive Thoughts Scale: instrument constructed by developing items according to Beck's cognitive triad (Beck, Rush, Shall & Emery, 1982) and other belief assessment instruments that were among the most used in scientific articles and/or that had in their content items that assessed thoughts related to depression based on the triad (Crandell Instrument on the Cognitive Triad; Automatic Thoughts Questionnaire; Attributional Style Questionnaire; Expanded Attributional Style Questionnaire; Beliefs Scale; Cognitive Triad Inventory; Irrational Values ​​Scale –IVS; Mood Survey; Self Rating Depression Scale SDS; Children Cognitive Assessment Questionnaire –CCAQ; Dysfunctional Attitude Scale -DAS). After the development of items by the authors of the project and the translation of items contained in other scales that assessed depression, those that related to beliefs of incapacity were maintained, related by A. T. Beck (1982) as those related to feelings of self-recrimination, guilt, feelings of uselessness, and low self-esteem in the face of external issues (the subject's relationship with others and the world). The final version of the instrument has 26 items, divided into two factors, the first being low self-esteem/hopelessness, with 16 items referring to the subject's negative perception of themselves and their future prospects, and the second on functionality in relationships, with 10 items on the subject's positive assessment of their relationship with others and how they evaluate the support they receive. The scale has a reliability of 0.93, with 0.93 being Factor 1 and 0.89 being Factor 2. For counting, the higher the score, the greater the dysfunctional thoughts.

Beck Depression Inventory second edition – BDI II (Goreinstein, Pang, Argimon & Werlang, 2011): validated for Brazil, it consists of 21 items and measures the intensity of depression in both diagnosed patients and probable cases in the normal population between the ages of 13 and 59. It is a four-point Likert scale, in which the higher the score, the greater the level of depression in the last 14 days. This instrument has some differences from the first version, such as the item that assesses loss of appetite, insomnia and social withdrawal, which were reworked according to the 4th edition of the DSM IV (APA, 1994). Four items were removed from the first version (weight loss, change in self-image, somatic perceptions and difficulty working) and replaced by agitation, devaluation, difficulty concentrating and lack of energy. The instrument has 21 items, and the higher the score, the greater the intensity of depression.

Generalized Anxiety Disorder 7 - GAD 7 (Spitzer, Kroenke, Williams, & Löwe, 2006): It consists of a brief, self-report instrument that measures the frequency of anxiety symptoms in the last two weeks. Developed with seven items and with a score from 0 to 3, the total score is obtained from the sum of the items, ranging from 0 to 21 points. Participants are classified into groups according to their level of anxiety, 0-4 as minimum, 5-9 mild, 10-14 moderate, and 15-21 severe. In the Portuguese version, the scale demonstrated good reliability; both the Cronbach's alpha coefficient (α = 0.916) and rho composite reliability coefficient (ρ = 0.909) were adequate. (Moreno et al., 2016).

Global Assessment of Functioning Scale - GAF: The GAF is a functioning assessment scale that ranges from 0 to 100, with higher scores indicating higher levels of functioning.

Clinical Global Impression Scale - CGI (Busner & Targum, 2007): The CGI is a physician-determined summary measure that takes into account all available information, including knowledge of the patient's history and the impact of symptoms on the patient's ability to function. It comprises two components, the Clinical Global Impression – Severity Scale (CGI-S), which assesses the severity of the illness, with scores ranging from 1 (normal, not ill) to 7 (extremely severe mental illness), and the Clinical Global Impression – Improvement Scale (CGI-I), which assesses the overall improvement since the start of treatment, with scores ranging from 1 (much better) to 7 (much worse).

Behavioral Activation for Depression Scale Short Form (BADS-SF) (Manos et al., 2011) : The BADS is a scale developed based on the behavioral model of depression. It aims to measure behavioral aspects that are correlated with clinical changes. It has two versions, an extended version and a brief version with 25 and 9 items, respectively. Each item is measured on a scale of 0 to 6 points, divided into two factors: activation and avoidance. The 9-item BADS-SF, validated for the Brazilian population, will be used in the study (Aschar et al. 2021).

ShapsBr-Snaith-Hamilton Pleasure Scale (Sanith et. al, 1995): SHAPS Pleasure Scale is an instrument used to assess the experience of pleasure or the anticipation of a pleasurable experience. This scale consists of 14 items, in which participants indicate their level of agreement on a four-point scale: "definitely agree," "agree," "disagree," and "definitely disagree." A higher score indicates a greater level of agreement. anhedonia. Validity in the Portuguese version by Jesus-Nunes et. al (2021), presented internal consistency of the instrument, evaluated by Cronbach's alpha coefficient, demonstrated to be adequate (α = 0.759; n = 228; 14 items).

Young Mania Rating Scale (YMRS) (Young et al., 1978): scale composed of 11 items, scored from 0 to 4 and from 0 to 4 (affirmability, speech, thought content and disruptive-aggressive behavior, scored twice to compensate for the patient's lack of cooperation). It is the most used scale in clinical studies with manic patients, with a high reliability index among avulsers (around 0.93 for the total score and between 0.66 * 0.92 for the individual items). For Young, the version translated by Vilela (2000) was adopted, with a cut-off point <7 as indicative of significant symptoms of mania/hypomania.

**4.4 Procedures**

This study will be conducted longitudinally to investigate the impact of a psychoeducation program for depression. To this end, psychiatric patient participants must meet the inclusion criteria, which necessarily indicate the presence of a Major Depressive Episode/Disorder or Recurrent Depressive Disorder without psychotic symptoms. Patients will be allocated to one of two groups (control or experimental) by simple randomization (1:1) that will be performed through the website www.sealedenvelope.com . This website generates blocks of random sizes and lengths.

The study will be conducted using the steps described above, but it is worth reiterating them. Patients will be admitted to the study after the inclusion criteria have been met, the project has been approved by the institution's ethics committee, and the subjects have given their consent. Testing will be done using instruments to assess the severity of depression symptoms (HAM-D), thoughts (EPD), avoidance behaviors (CBAS and BADS), levels/intensity of depression (BDI II), and an identification questionnaire. To better understand the impact of the psychoeducational approach, we sought to include other instruments that would facilitate this interpretation, such as the CGI and GAF ​​for impacts on quality of life, anxiety (GAD-7), and experience of pleasure (Shaps). The duration of the application of the instruments will depend on the phases, with the time stipulated for the application of the clinical group being 1 hour and 40 minutes, including the SCID. For psychoeducation, the meetings should last 90 minutes, occurring weekly over six weeks (see Table 1).

**Usual treatment (TAU):** Treatment will be initiated with Sertraline (SSRI) – doses of 50 mg and adjusted as necessary according to the patient's profile. Dosage adjustments will be made according to the scores on the HAMD and Udvalg for Kliniske Undersøgelser (UKU) side effect assessment scale (Lingjaerde et al., 1987). After 8 weeks, if the patient presents remission, he/she will continue on monotherapy with sertraline. In case of relapse, Bupropion, Quetiapine, Lithium or Aripiprazole may be complementary. In this case, the complementary medications will be determined according to the patient's profile by the physician. If there is a response of more than 25% of the symptoms, but without remission, the patient will proceed to potentiation with the aforementioned medications and if there is no improvement of at least 25%, the patient will be considered to have withdrawn. Combinations with Lorazepam, if necessary, will be allowed at any stage of the study.

**Psychoeducation program implementation plan:** The sessions will initially be set up with approximately 10 minutes of introductory time (in which free topics will be discussed). The following 40 minutes will be dedicated to presenting the proposed topics in interactive class plans using slides, and a printed version of the same material will be provided. Next, 20 minutes will be reserved for discussion and training on the information provided, and the remaining 20 minutes will be reserved for reviewing what was discussed and presented, as well as for questions and clarifications. The sessions will be held in person and open, allowing new members to start at any of the sessions. For participants in the control group who choose to receive psychoeducation, the structure will follow the same.

Below is a description of the topics covered in each session.

**Table 1. Psychoeducation sessions**

| **Session** | **Topics** |
| --- | --- |
| **1. What is depression?** | - Explanation to participants about the nature and clinical characteristics of depressive disorder; - Differentiate sadness from depression; - Signs and symptoms; |
| **2. Causes and treatment** | - Causes (biological, psychological and environmental factors); - Presentation on which treatments are recommended for depression; - Pharmacological treatment and other forms of treatment. |
| **3. Identifying risk factors and dealing with crises** | - What factors contribute to depression not improving? - What to do during a crisis, what strategies can help. - Identifying depressive symptoms and what to do when a new episode is detected? - Management methods after identifying a new episode. |
| **4. Benefits of adopting a regular lifestyle** | - The role of lifestyle in depression; - Importance of the circadian cycle and sleep hygiene techniques. |
| **5. Troubleshooting** | - Communication styles – developing assertiveness - Making decisions. |
| **6. Practical strategies** | - Fact checking (cognitive strategy); - Accumulating positive emotions (importance of pleasurable activities); - Mindfulness (importance of being focused on the present moment, strategies for observing, describing and participating). |

**5. Work Plan and Schedule for its Execution**

**Table 2. Scheme of application of scales throughout the project**

|  | **V1** | **V2** | **After 3 months** | **After 6 months** | **After 12 moths** |
| --- | --- | --- | --- | --- | --- |
| **SCID** |  |  |  |  |  |
| **HAMD** |  |  |  |  |  |
| **BADS** |  |  |  |  |  |
| **SHARPS** |  |  |  |  |  |
| **CGI** |  |  |  |  |  |
| **GAF** |  |  |  |  |  |
| **GAD-7** |  |  |  |  |  |
| **CBAS** |  |  |  |  |  |
| **BDI II** |  |  |  |  |  |
| **EPD** |  |  |  |  |  |
| **Young** |  |  |  |  |  |
| **Identification questionnaire** |  |  |  |  |  |
| **Knowledge questionnaire** |  |  |  |  |  |

**Caption:** BDI II - Beck Depression Rating Scale, CBAS - Cognitive Behavioral Avoidance Scale, EPD - Depressive Thoughts Scale, GAD - Generalized Anxiety Disorder, GAF - Global Assessment of Functioning Scale, SCID - Structured Interview for DSM IV, SHARPS - ShapsBr-Snaith-Hamilton Pleasure Scale V1. Entry, before intervention; V2. End of the sixth psychoeducation session

**6. Data Analysis Plan**

Initially, a descriptive analysis will be performed on sociodemographic data, impact of psychoeducation and severity of symptoms. Considering that the data will allow considering the data as parametric, the main outcome (reduction in severity of symptoms) will be evaluated by Student's t-tests for mean difference, ANOVA and MANOVA of dependent [pre and post] and independent [GC and GE] samples. All analyses will be performed using the STATA BE 18 program, and data will be considered statistically significant when p is equal to or greater than 0.05.

**7. Timeline**

The project is expected to last approximately 48 months. Below is the expected timeline for the project.

|  | **Year 1** | **Year 2** | **Year 3** | **Year 4** |
| --- | --- | --- | --- | --- |
| **Data writing** |  |  |  |  |
| **Updating bibliographic references** |  |  |  |  |
| **Data collection** |  |  |  |  |
| **Statistical analysis** |  |  |  |  |
| **Manuscript preparation** |  |  |  |  |
| **Publication** |  |  |  |  |

**References**

American Psychiatric Association. Diagnostic and Statistical Manual of Mental Disorders, Fifth Edition (DSM-5-TR). Arlington: American Psychiatric Publishing; 2022.

Beesdo K, Bittner A, Pine DS, et al. Incidence of social anxiety disorder and the consistent risk for secondary depression in the first three decades of life. Arch Gen Psychiatry 2007; 64: 903–12.

Bhattacharjee, D., Rai, A. K., Singh, N. K., Kumar, P., Munda, S. K., & Das, B. (2011). Psycho-education: A measure to strengthen psychiatric treatment. *Delhi Psychiatric Journal*, *14*(1),33-39.

Bockting, C.L., et al., A lifetime approach to major depressive disorder: The contributions of psychological interventions in preventing relapse and recurrence, Clinical Psychology Review (2015).

Busner J, Targum SD. The clinical global impressions scale: applying a research tool in clinical practice. *Psychiatry (Edgmont)*. 2007;4(7):28-37.

Carneiro, A.M.. Pensamentos disfuncionais e comportamentos evitativos ao longo do episódio depressivo: um estudo longitudinal. 2019. 154 f. Tese (Doutorado) - Faculdade de Medicina, Universidade de São Paulo, São Paulo, 2019.

Carneiro, A. M.; Dobson, K. S. Cognitive-behavioral treatment for major depressive disorder: a narrative review. Revista Brasileira de Terapias Cognitivas, v. 12, n. 1, 2016.

Cheng C, Cheung MW, Montasem A; 44 Members of the International Network of Well-Being Studies. Explaining differences in subjective well-being across 33 nations using multilevel models: universal per- sonality, cultural relativity, and national income. J Pers. 2016;84:46-58.

Del Porto J.A, Sarin LM, Moriyama T.S. Depressões resistentes. In: Lacerda ALT de, Quarantini L de C, Miranda-Scippa AMA, Del Porto JA. Depressão: do neurônio ao funcionamento social. Porto Alegre: Artmed; 2009. p. 163-179.

Fleck MP, Berlim MT, Lafer B, et al. (2009) Review of the guidelines of the Brazilian Medical Association for the treatment of depression. Revista Brasileira de Psiquiatria 31: S7–17.

Gutiérrez-Rojas L, Porras-Segovia A, Dunne H, Andrade-González N, Cervilla JA. Prevalence and cor- relates of major depressive disorder: a systematic review. Braz J Psychiatry. 2020;42:657-672. http://dx.doi.org/10.1590/1516-4446- 2019-0650

Jesus-Nunes AP, Coroa JPBB, Argolo FC, et al. Psychometric properties the of Brazilian Portuguese version of Snaith-Hamilton Pleasure Scale (SHAPS). *Trends Psychiatry Psychother*. 2021;43(1):23-29. doi:10.47626/2237-6089-2019-0066

Kessler RC. Epidemiology of women and depression. J Affect Dis- ord. 2003;74:5-13.

Kessler, R.C., Berglund, P., Demler, O., Jin, R., Merikangas, K.R., & Walters, E.E. (2005). Life- time Prevalence and Age-of-Onset Distributions of DSM-IV Disorders in the National Comorbidity Survey Replication. Archives of General Psychiatry, 62(6), 593–602. http://dx.doi.org/10.1001/archpsyc.62.6.593.

Luppino FS, de Wit LM, Bouvy PF, et al. Overweight, obesity, and depression: a systematic review and meta-analysis of longitudinal studies. Arch Gen Psychiatry 2010; 67: 220–29.

Manos, Rachel C., et al. “The Behavioral Activation for Depression Scale–Short Form: Development and Validation.” Behavior Therapy, vol. 42, no. 4, Dec. 2011, pp. 726–739, https://doi.org/10.1016/j.beth.2011.04.004

Moffitt TE, Harrington H, Caspi A, et al. Depression and generalized anxiety disorder: cumulative and sequential comorbidity in a birth cohort followed prospectively to age 32 years. Arch Gen Psychiatry 2007; 64: 51–60.

Moreno, A. L., Sousa, D. A., Souza, A. M. F. L. P, Manfro, G. G., Salum, G. A., Koller, S. H., Osório, F. L., Crippa, J. A. de S. (2016). Factor structure, reliability, and item parameters of the Brazilian-portuguese version of the GAD-7 questionnaire. Temas em Psicologia, 24(1), 367–376. https://doi.org/10.9788/TP2016.1-25

Rozenthal M, Laks J, Engelhardt E. Aspectos neuropsicológicos da depressão. Revista de Psiquiatria do Rio Grande do Sul. 2004 26:204-12.

Spitzer RL, Kroenke K, Williams JB, Löwe B. A brief measure for assessing generalized anxiety disorder: the GAD-7. *Arch Intern Med*. 2006;166(10):1092-1097. doi:10.1001/archinte.166.10.1092

Srinivasan, J., Cohen, N. L., & Parikh, S. V. (2003). *Patient Attitudes regarding Causes of Depression: Implications for Psychoeducation. The Canadian Journal of Psychiatry, 48(7), 493–495.*

Talkovsky AM, Green KL, Osegueda A, Norton PJ. Secondary depression in transdiagnostic group cognitive behavioral therapy among individuals diagnosed with anxiety disorders. J Anxiety Disord. 2017;46:56-64.

Tursi-Braga, MFS. Eficácia da psicoeducação para pacientes com depressão unipolar (dissertação). Ribeirão Preto: Universidade de São Paulo, Faculdade de Medicina, 2014. 137f.

World Health Organization (WHO). (2016). Fact sheet depression. Recuperado de: <http://www.who.int/mediacentre/factsheets/fs369/en/>.

Young R, Biggs J, Ziegler V. Meyer D. A rating scale for mania reliability, validity and sensibility. Br J Psychiatry, 1978,133-429-35.
